# Targeting fungal membrane homeostasis with imidazopyrazoindoles impairs azole resistance and biofilm formation

**DOI:** 10.1038/s41467-022-31308-1

**Published:** 2022-06-25

**Authors:** Nicole M. Revie, Kali R. Iyer, Michelle E. Maxson, Jiabao Zhang, Su Yan, Caroline M. Fernandes, Kirsten J. Meyer, Xuefei Chen, Iwona Skulska, Meea Fogal, Hiram Sanchez, Saif Hossain, Sheena Li, Yoko Yashiroda, Hiroyuki Hirano, Minoru Yoshida, Hiroyuki Osada, Charles Boone, Rebecca S. Shapiro, David R. Andes, Gerard D. Wright, Justin R. Nodwell, Maurizio Del Poeta, Martin D. Burke, Luke Whitesell, Nicole Robbins, Leah E. Cowen

**Affiliations:** 1 Department of Molecular Genetics, University of Toronto, Toronto, ON Canada; 2 Program in Cell Biology, The Hospital for Sick Children, Toronto, ON Canada; 3 Department of Chemistry, Roger Adams Laboratory, University of Illinois at Urbana-Champaign, Urbana, IL USA; 4 Carl R. Woese Institute for Genomic Biology, University of Illinois at Urbana-Champaign, Urbana, IL USA; 5 Department of Microbiology and Immunology, Stony Brook University, Stony Brook, NY USA; 6 Department of Biochemistry, University of Toronto, Toronto, ON Canada; 7 David Braley Centre for Antibiotics Discovery, M.G. DeGroote Institute for Infectious Disease Research, Department of Biochemistry and Biomedical Sciences, McMaster University, Hamilton, ON L8S 4K1 Canada; 8 Department of Molecular and Cellular Biology, University of Guelph, Guelph, ON Canada; 9 Department of Medicine, University of Wisconsin School of Medicine and Public Health, Madison, WI USA; 10 Department of Medical Microbiology and Immunology, University of Wisconsin, Madison, WI USA; 11 Department of Molecular Genetics, Donnelly Centre for Cellular and Biomolecular Research, Toronto, ON Canada; 12 RIKEN Center for Sustainable Resource Science, Wako, Saitama, Japan; 13 Department of Biotechnology, Graduate School of Agricultural Life Sciences, The University of Tokyo, Tokyo, Japan; 14 Collaborative Research Institute for Innovative Microbiology, The University of Tokyo, Tokyo, Japan; 15 Division of Infectious Diseases, Stony Brook University, Stony Brook, NY USA; 16 Veteran Administration Medical Center, Northport, NY USA; 17 Department of Biochemistry, Roger Adams Laboratory, University of Illinois at Urbana-Champaign, Urbana, IL USA; 18 Department of Microbiology, University of Illinois at Urbana-Champaign, Urbana, IL USA

**Keywords:** Fungi, Fungal infection, High-throughput screening, Antifungal agents

## Abstract

Fungal infections cause more than 1.5 million deaths annually. With an increase in immune-deficient susceptible populations and the emergence of antifungal drug resistance, there is an urgent need for novel strategies to combat these life-threatening infections. Here, we use a combinatorial screening approach to identify an imidazopyrazoindole, NPD827, that synergizes with fluconazole against azole-sensitive and -resistant isolates of *Candida albicans*. NPD827 interacts with sterols, resulting in profound effects on fungal membrane homeostasis and induction of membrane-associated stress responses. The compound impairs virulence in a *Caenorhabditis elegans* model of candidiasis, blocks *C. albicans* filamentation in vitro, and prevents biofilm formation in a rat model of catheter infection by *C. albicans*. Collectively, this work identifies an imidazopyrazoindole scaffold with a non-protein-targeted mode of action that re-sensitizes the leading human fungal pathogen, *C. albicans*, to azole antifungals.

## Abstract (web summary)

There is an urgent need for novel strategies to combat life-threatening fungal infections. Here, Revie et al. identify a compound that induces alterations in fungal membranes, synergizes with azole antifungals against the pathogen *Candida albicans*, and inhibits virulence traits and biofilm formation in animal models of infection.

## Introduction

In recent decades, modern medicine has revolutionized the treatment of numerous human diseases through the use of immunomodulatory and invasive medical interventions. As a consequence, opportunistic fungal pathogens have become a leading cause of human morbidity and mortality in vulnerable, immune-compromised populations^1,2^. Invasive fungal infections are responsible for global mortality rates that exceed that of malaria or breast cancer, with over 1.5 million deaths per year worldwide^1,3^. *Candida* species are a major contributor to these infections, with *Candida albicans* reigning as the fourth most common cause of hospital-acquired bloodstream infections, with mortality remaining at ~40%^1,4^. This underappreciated public health threat has been met with the overuse of a limited arsenal of antifungals for prophylactic purposes, leading to the widespread emergence of multidrug-resistant pathogenic fungi^5^.

In contrast to approximately two dozen classes of antibacterials in current clinical use, only three major classes of antifungals are approved for the treatment of invasive fungal infections^6^. Fungistatic azoles have been the most widely deployed treatment for systemic candidiasis for decades and target the ergosterol biosynthetic enzyme lanosterol 14α-demethylase, encoded by *ERG11*^7^. Analogous to cholesterol in mammalian cells, ergosterol is critical for maintaining fungal cell membrane integrity and fluidity^7^. Azole-induced depletion of ergosterol from the cell is accompanied by accumulation of alternative sterol intermediates that perturb membrane stability and arrest fungal growth^7^. The pleiotropic effects on membrane homeostasis induced by azoles include severe defects in vacuolar integrity and impairment of vacuole–vacuole fusion^8^. Widespread overuse of the fungistatic azoles has led to the emergence of various mechanisms of resistance, including modification and/or overexpression of the drug target, upregulation of drug-efflux pumps, and compensatory alterations within the ergosterol biosynthesis pathway^2^. Moreover, lipidomic analysis of azole-resistant isolates has revealed alterations in both sterols and sphingolipids, key membrane components that regulate membrane fluidity and stabilize membrane-resident proteins^9,10^. Given the increasing frequency of resistance, an urgent need exists for the development of novel therapeutic strategies that can be used in combination with current treatments and restore their efficacy.

Despite decades of effort, progress in the development of novel antifungal agents has been modest. Evidence from the model yeast *Saccharomyces cerevisiae* suggests that antifungal therapeutic strategies need to take into account the highly interconnected and functionally redundant genetic networks that underpin fungal biology^11,12^. This genetic buffering partly explains the inherent difficulty of identifying single compounds that possess fungal-selective cytotoxicity and advocates for the development of combination therapies^13,14^. Combination therapy has the potential to confer enhanced efficacy, more selective fungal-toxicity, and reduced vulnerability to the evolution of drug resistance^15,16^. While combination therapy has been underutilized in the treatment of most invasive fungal diseases, the combination of the polyene antifungal amphotericin B with the anti-metabolite 5-flucytosine is the current gold standard for cryptococcal meningitis, a fungal infection that afflicts roughly 220,000 annually^17^. The benefit of the combination in preventing the rapid emergence of resistance to 5-flucytosine serves as proof-of-principle and highlights the promising, but relatively untapped potential of combination strategies^18,19^.

In this work, we screened ~20,000 compounds from the RIKEN Natural Product Depository (NPDepo), a collection of natural products and their derivatives, in combination with a minimally inhibitory concentration of the conventional antifungal fluconazole to identify combinations with enhanced activity against *C. albicans*. This screen identified several compounds sharing an imidazopyrazoindole scaffold that strongly synergize with fluconazole against diverse fungal species, especially azole-resistant isolates of *C. albicans*. Using chemical-genetic, biochemical, and biophysical approaches, we establish a relatively unusual mode of action for the most active hit, NPD827, which involves disruption of membrane homeostasis rather than inhibition of a specific protein target. With promising efficacy in diverse preclinical models of *C. albicans* infection, NPD827 provides a promising lead compound for further development of a combination treatment approach to block key fungal virulence traits and re-sensitize resistant fungal isolates to azoles.

## Results

### Identification of imidazopyrazoindoles with azole-potentiating activity

**Fig. 1:**
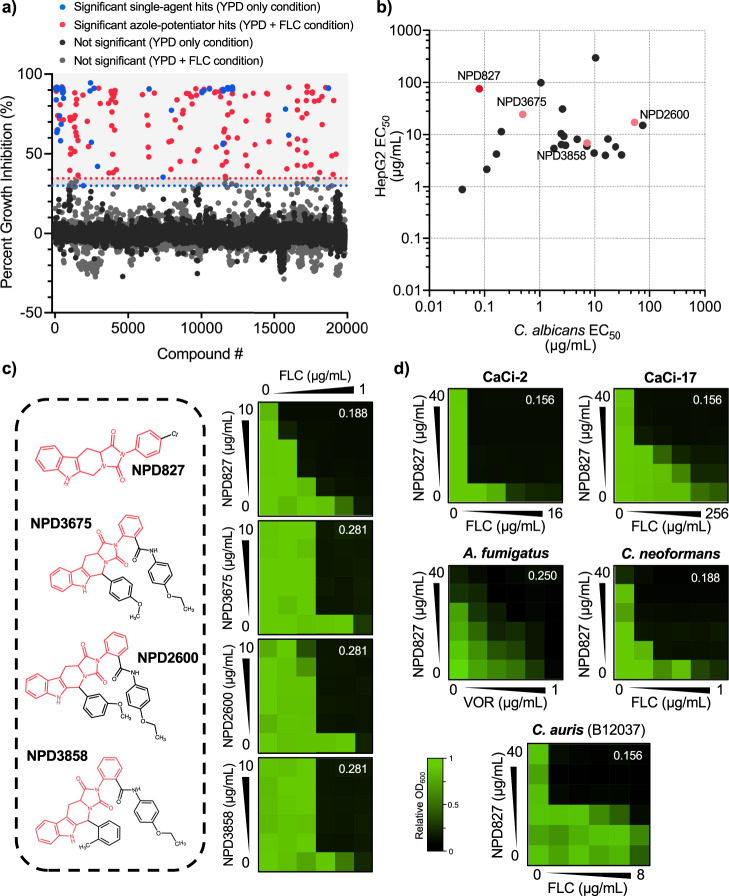
RIKEN Natural Product Depository screen against *C. albicans* identifies compounds that potentiate azole activity. **a** Screening data are reported as percent growth inhibition (%) in the absence and presence of fluconazole (FLC, 1 μg/mL). Compounds are colored based on classification as single agent-active (blue, ≥20-MAD above median growth inhibition), or azole potentiator (red, ≥20-MAD above median growth inhibition with fluconazole). **b** Fungal versus human growth inhibition for the top 24 compounds identified in the primary screen. EC_50_ values were calculated by nonlinear regression curve fitting of triplicate determinations from dose–response assays against human cells (HepG2) and *C. albicans* (CaSS1). All assays were performed in the presence of 0.25 µg/mL of FLC. Pink data points indicate compounds belonging to the imidazopyrazoindole class. Red data point highlights NPD827. **c** Checkerboard assays with four imidazopyrazoindoles in combination with FLC. Checkerboards were performed in YPD medium at 30 °C against *C. albicans* (CaSS1) and growth was measured using absorbance at 600 nm after 24 h. Technical duplicates were averaged, and measurements were normalized to no drug controls. Relative growth is quantitatively represented by color (see scale bar at bottom). Compound structures are depicted on the left and checkerboard heatmaps are depicted on the right. Fractional Inhibitory Concentration Index (FICI) values are displayed in the top right corner of each checkerboard (FICI < 0.5, synergistic interaction). **d** Checkerboard assays were performed with NPD827 and either fluconazole (FLC) or voriconazole (VOR) against *C. albicans* azole-tolerant and azole-resistant clinical isolates (CaCi-2 and CaCi-17, respectively), as well as against the evolutionary divergent fungal pathogens *C. auris, C. neoformans*, and *A. fumigatus*. Checkerboards were performed as described in (**c**). After 24 h (for *C. albicans* and *C. auris*) or 48 h (for *C. neoformans, A. fumigatus*), growth was measured and plotted as in (**c**). Source data are provided as a Source Data file.

To explore combinatorial antifungal chemical space, we screened the RIKEN NPDepo chemical library, a collection of natural products and their derivatives, in the absence and presence of fluconazole (1 µg/mL) against a wild-type strain of *C. albicans*. Compounds that reduced growth after 24 h by 20-median absolute deviations (MADs) from the median alone were classified as single-agent hits. Compounds that reduced growth after 24 h by 20-MADs in the presence of fluconazole compared to no-fluconazole controls (% fluconazole potentiation) were classified as potentiators. Of 19,840 compounds screened at 10 µg/mL, we identified 56 single agents (hit rate: 0.28%) and 151 fluconazole potentiators (hit rate: 0.76%) (Fig. 1a), highlighting the potential of screening compounds in combination to dramatically expand the antifungal space. The bioactivity of compounds that could be independently sourced was verified through standard dose–response assays, yielding 59 compounds with confirmed azole-potentiating activity at or below their screening concentration. Hits were further filtered based on the magnitude of their synergistic interaction with fluconazole, with 24 molecules displaying synergy against *C. albicans* (Fractional Inhibitory Concentration Index [FICI] < 0.5; Table 1). Finally, hits were prioritized by comparing their EC_50_ values against the human liver cancer-derived cell line, HepG2, to their EC_50_ values against *C. albicans*. A total of 14 compounds were at least two-fold more potent against *C. albicans* in the presence of 0.25 µg/mL of fluconazole compared to their potency against HepG2 cells in culture (Fig. 1b). Interestingly, this analysis identified a shared imidazopyrazoindole core in four prioritized molecules with a maximum common substructure (MCS) Tanimoto score of >0.5 (Fig. 1c). The compound NPD827 was chosen for follow-up given its potent activity against *C. albicans* in the presence of fluconazole (EC_50_ < 0.1 µg/mL; Fig. 1b) and minimal cytotoxicity against HepG2 cells (EC_50_ > 80 µg/mL; Fig. 1b).

**Table 1:** Top 24 azole potentiators displaying a synergistic interaction with fluconazole.

Compound ID	FICI	Compound ID	FICI
NPD13393	0.268	NPD2864	0.258
NPD827	0.188	NPD3363	0.258
NPD3675	0.258	NPD3155	0.375
NPD13211	0.258	NPD7295	0.313
NPD3774	0.258	NPD4066	0.281
NPD1752	0.281	NPD2887	0.258
NPD2470	0.258	NPD2955	0.258
NPD2889	0.313	NPD4138	0.258
NPD3126	0.455	NPD2756	0.281
NPD3540	0.266	NPD3858	0.258
NP771	0.250	NPD4407	0.258
NPD2396	0.258	NPD2600	0.258

Synergistic interactions were determined by performing checkerboard assays with each NPD compound and fluconazole (FLC) against wild type *C. albicans* (SN95). Growth in checkerboard assays were measured using absorbance at 600 nm after 24 h at 30 °C. Synergistic interactions were quantified by calculating the Fractional Inhibitory Concentration Index (FICI) for each compound pair. FICI is calculated as: FICI = (MIC_90_ NPD in combination/MIC_90_ NPD alone) + (MIC_90_ FLC in combination/MIC_90_ FLC alone), where MIC_90_ represents the minimal inhibitory concentration at 90% growth inhibition.

To further assess the antifungal activity of NPD827, we performed checkerboard assays in combination with fluconazole against two clinical isolates of *C. albicans*. These isolates included an azole-tolerant strain from an HIV-infected patient recovered early in the course of a persistent *Candida* infection (CaCi-2) and an azole-resistant isolate recovered from the same patient after multiple treatments with fluconazole (CaCi-17)^20^. NPD827 was synergistic with fluconazole against both CaCi-2 and CaCi-17 (Fig. 1d), highlighting the ability of this compound to augment azole activity despite overexpression of efflux transporters and the presence of resistance-conferring mutations in the azole target gene *ERG11*^20^. This observation was further supported by performing checkerboard assays against diverse laboratory-derived azole-resistant isolates as well as additional clinical isolates. In all cases NPD827 enhanced azole efficacy (Supplementary Fig. 1a). To assess the antifungal spectrum of combination treatment, checkerboard assays were performed against isolates of other non-*albicans Candida* species (*C. glabrata* and *C. auris*), the basidiomycete *Cryptococcus neoformans*, and the filamentous mold *Aspergillus fumigatus*. NPD827 enhanced azole activity against select *C. auris* isolates, *C. neoformans*, and *A. fumigatus*, but displayed no activity against either of the two *C. glabrata* strains examined (Fig. 1d, Supplementary Fig. 1b, c).

**Fig. 2:**
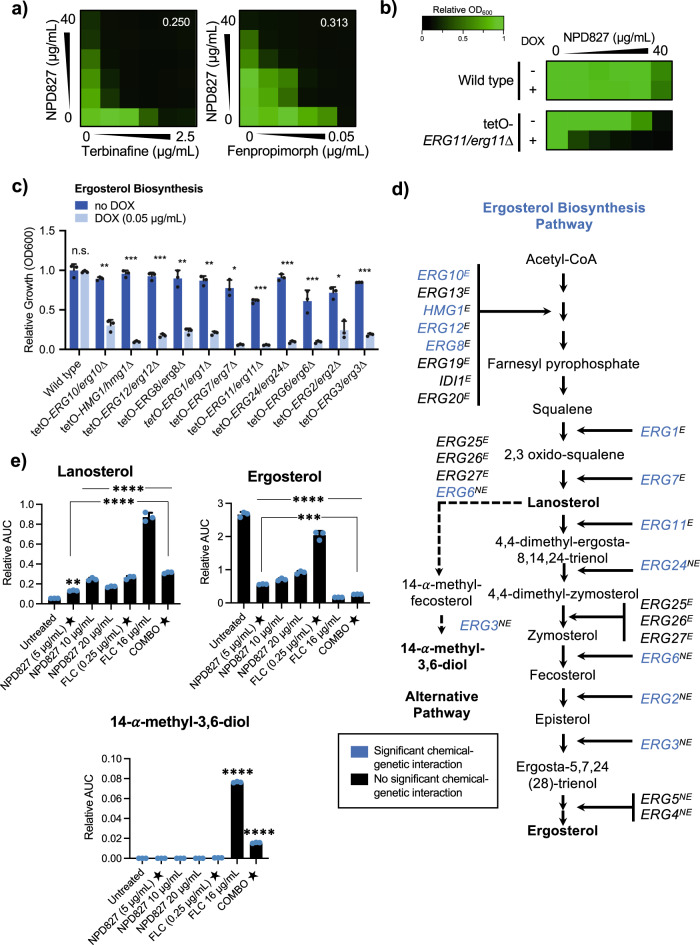
NPD827 displays chemical–genetic interaction with ergosterol biosynthesis genes and increases membrane sterol levels in combination with fluconazole. **a** Checkerboard assays were performed against wild type *C. albicans* (SN95) using NPD827 and the ergosterol biosynthesis inhibitors, terbinafine and fenpropimorph. Assays were performed as previously described in Fig. 1c. FICI values are displayed in the top right corner of each checkerboard. **b** Dose–response assays were performed against a wild-type and a conditional expression strain of *ERG11* (tetO-*ERG11/erg11Δ*) in the absence and presence of doxycycline (DOX, 0.05 µg/mL). Growth was measured using absorbance at 600 nm in YPD medium at 30 °C after 24 h. Relative growth is quantitatively represented by color (see bar for scale). **c** Conditional expression mutants of ergosterol biosynthesis pathway components were profiled at a fixed concentration of NPD827 (5 μg/mL) in the absence and presence of doxycycline (DOX, 0.05 μg/mL). Data are shown for those strains with significant differences, presented as mean ± SD of technical triplicates. *P*-value: 0.00279 (tetO-*ERG10/erg10Δ*), 0.00042 (tetO-*HMG1/hmg1Δ)*, 0.00024 (tetO-*ERG12/erg12Δ*), 0.00345 (tetO-*ERG8/erg8Δ*), 0.00088 (tetO-*ERG1/erg1Δ*), 0.00644 (tetO-*ERG7/erg7Δ*), 0.00045 (tetO-*ERG11/erg11Δ*), 0.00019 (tetO-*ERG24/erg24Δ*), 0.02180 (tetO-*ERG6/erg6Δ*), 0.00603 (tetO-*ERG2/erg2Δ*), 0.00022 (tetO-*ERG3/erg3Δ*). Significance was determined by performing multiple unpaired *t* tests comparing plus DOX conditions for each strain with no DOX; *p*-value: *<0.05, **<0.005, ***<0.0005. **d** Overview of ergosterol biosynthesis pathway labeled with gene names for the conditional expression mutants that were tested for sensitivity to NPD827. E denotes essential genes and NE denotes non-essential genes. Genes coloured in blue were significantly hypersensitive to NPD827 upon transcriptional repression with DOX. **e** Relative abundance of lanosterol, ergosterol, and 14-α-methyl-3,6-diol was determined in *C. albicans* (SN95) after compound treatment (star indicates concentrations used in combination treatment) relative to internal cholesterol standard. Data are presented as mean ± SD of triplicate runs. Significance was determined by one-way ANOVA with Bonferroni’s multiple comparisons test; *p*-value: ***< 0.001, ****<0.0001. *p*-value, compared to untreated: Lanosterol, 0.0042 (NPD827 5 µg/mL); Lanosterol, <0.0001 (NPD827 10 µg/mL, NPD827 20 µg/mL, FLC 0.25 µg/mL, FLC 16 µg/mL, Combination [FLC 0.25 µg/mL, NPD827 5 µg/mL]); *p*-value compared to NPD827 5 µg/mL, <0.0001 (Combination); Ergosterol, compared to untreated <0.0001 (all samples); *p*-value compared to NPD827 5 µg/mL, 0.0006 (Combination); 14-α-methyl-3,6-diol, <0.0001 (FLC 16 µg/mL, Combination [FLC + NPD827). Source data are provided as a Source Data file.

To learn whether NPD827 might enhance the activity of other ergosterol biosynthesis inhibitors, we performed checkerboard assays with NPD827 and terbinafine, which targets Erg1^21^, and fenpropimorph, which targets Erg2 and Erg24^22^. NPD827 enhanced the activity of both these ergosterol biosynthesis inhibitors against a wild-type strain of *C. albicans* (Fig. 2a). This activity appeared restricted to compounds which impair sterol biosynthesis because NPD827 had an antagonistic interaction with amphotericin B, a polyene that extracts ergosterol from the fungal cell membrane (Supplementary Fig. 1d)^23^. Further, NPD827 showed no chemical–chemical interaction with the cell wall-targeting antifungal, caspofungin (Supplementary Fig. 1d). Thus, NPD827 selectively enhances the activity of inhibitors of the ergosterol biosynthesis pathway.

### NPD827 perturbs sterol and sphingolipid homeostasis

Finding that NPD827 potentiates fluconazole activity, we investigated whether depletion of Erg11, the target of azoles, would sensitize *C. albicans* to NPD827. To do this, we employed a conditional expression mutant, where one allele of *ERG11* is deleted and the remaining allele is controlled by a tetracycline-repressible promoter^24^. Transcriptional repression of *ERG11* with the tetracycline analog doxycycline (DOX) caused significant hypersensitivity to NPD827 relative to the no-DOX control (Fig. 2b). We expanded this analysis to other components of the ergosterol biosynthesis pathway and found that depletion of many, but not all, caused significant hypersensitivity to NPD827. Specifically, transcriptional repression of several genes responsible for the generation of upstream sterol intermediates (*ERG10, HMG1, ERG12, ERG8, ERG1, ERG7, ERG11, ERG24, ERG6, ERG2*, and *ERG3*), caused hypersensitivity to NPD827 (Fig. 2c, d). This was consistent with our observation that NPD827 potentiated fenpropimorph and terbinafine activity (Fig. 2a). However, depletion of certain early (*ERG13, ERG19, IDI1, ERG20*) or late components (*ERG5, ERG4*) of the ergosterol biosynthesis pathway had no impact on NPD827 activity, for reasons that are unclear.

To examine the effect of NPD827 on sterol biosynthesis more directly, we used GC-MS to measure the abundance of select metabolites following treatment with NPD827, either in the absence or presence of fluconazole. As expected, a high-concentration of fluconazole (16 µg/mL) caused a marked decrease in ergosterol, a key constituent of the fungal cell membrane, and a corresponding increase in the immediate upstream substrate of Erg11 (lanosterol) as well as a toxic derivative (14-α-methyl-3,6-diol) that accumulates upon Erg11 inhibition (Fig. 2e). In the absence of fluconazole, NPD827 caused a significant depletion of ergosterol, and a moderate increase in lanosterol. In combination with fluconazole, ergosterol levels were modestly but significantly reduced relative to NPD827 treatment alone and 14-α-methyl-3,6-diol levels were significantly increased relative to individual treatments (Fig. 2e).

**Fig. 3:**
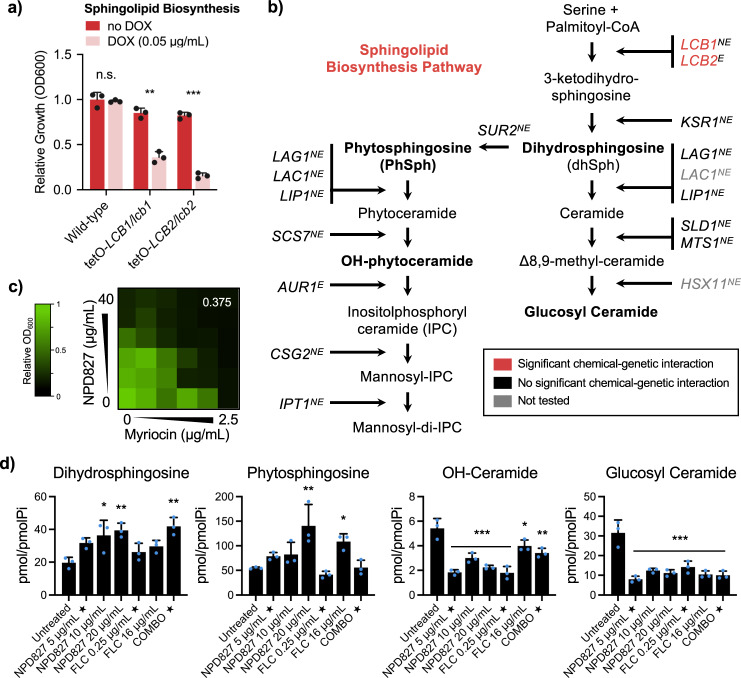
NPD827 treatment results in accumulation of long chain sphingoid bases and the depletion of modified ceramides. **a** Conditional expression mutants of sphingolipid biosynthesis pathway components were profiled at a fixed concentration of NPD827 (5 μg/mL) in the absence and presence of doxycycline (DOX, 0.05 μg/mL). Data are shown for those mutants with significant differences, presented as mean ± SD of technical triplicates. *p*-value: 0.00053 (tetO-*LCB1/lcb1Δ*), 0.00002 (tetO-*LCB2/lcb2Δ*). Significance was determined by performing multiple unpaired *t* tests comparing plus DOX conditions for each strain with no DOX. *p*-value: **<0.005, ***<0.0005. **b** Overview of sphingolipid biosynthesis pathway labeled with gene names of conditional expression mutants that were tested for sensitivity to NPD827. E denotes essential genes and NE denotes non-essential genes. Genes coloured in red were identified as significantly hypersensitive to NPD827 upon transcriptional repression with doxycycline (DOX, 0.05 μg/mL). **c** Checkerboard assays were performed with NPD827 and the SPT inhibitor myriocin against a wild-type strain of *C. albicans* (SN95). Assays were performed and are presented as previously described in Fig. 1c. FICI values are displayed in the top right corner of each checkerboard. **d** Abundance of key sphingolipid species with significant differences in compound-treated samples (star indicates concentrations used in combination treatment). Data are expressed as mean ± SD of *n* = 3, biologically independent replicates. *p*-value, compared to untreated: Dihydrosphingosine, 0.0086 (NPD827 10 µg/mL); 0.0023 (NPD827 20 µg/mL); 0.0008 (FLC 16 µg/mL); Phytosphingosine, 0.0009 (NPD827 20 µg/mL); 0.0313 (Combination [FLC 0.25 µg/mL + NPD827 5 µg/mL]); OH-Ceramide, <0.0001 (NPD827 5 µg/mL, 20 µg/mL, FLC 0.25 µg/mL); 0.0001 (NPD827 10 µg/mL); 0.0007 (FLC 16 µg/mL); 0.0102 (Combination [FLC + NPD827]); Glucosyl Ceramide, <0.0001 (all samples). Significance determined by one-way ANOVA with Bonferroni’s multiple comparisons test, where all treatment conditions were compared to the wild-type untreated condition. *p*-value: *<0.05, **<0.005, ***<0.0005. Source data are provided as a Source Data file.

Sterols are vital components of all eukaryotic membranes. In concert with sphingolipids, they maintain membrane integrity and participate in the formation specialized lipid rafts^25^. Furthermore, in yeast, alterations in membrane sterol levels often lead to compensatory changes in sphingolipid composition and organization^26^. Hence, we investigated the impact of NPD827 on sphingolipid metabolism. First, we assessed the sensitivity of an additional set of conditional expression mutants comprised of genes involved in the sphingolipid pathway. Transcriptional repression of two genes, *LCB1* and *LCB2*, encoding components of the serine palmitoyl transferase complex (SPT), conferred hypersensitivity to NPD827 (Fig. 3a, b). The SPT catalyzes the rate-limiting step of sphingolipid biosynthesis and regulates the production of sphingolipid long-chain bases (LCBs), excessive accumulation of which is toxic^27^. Consistent with the chemical–genetic interaction between NPD827 and SPT, NPD827 was synergistic with myriocin, a sphingolipid biosynthesis inhibitor that targets the SPT (Fig. 3c). Interestingly, genetic depletion of all other sphingolipid biosynthesis genes tested had no impact on NPD827 susceptibility (Fig. 3b). To determine whether sphingolipid levels in *C. albicans* are modulated by NPD827, we performed mass spectrometry-based lipidomic analysis of cells treated with NPD827 in the absence and presence of fluconazole. NPD827 treatment alone resulted in a significant dose-dependent increase in the LCBs, phytosphingosine (PhSph) and dihydrosphingosine (DhSph), and an overall reduction in key ceramide species, including OH-ceramide (OH-Cer), and glycosyl ceramide (GlcCer) (Fig. 3d). Notably, combining both NPD827 and fluconazole did not enhance effects on sphingolipid intermediate levels relative to either compound alone. Overall, we conclude that NPD827 acts in a way that perturbs lipid homeostasis at a relatively broad level.

### Perturbation of lipid homeostasis by NPD827 activates membrane-associated stress responses

**Fig. 4:**
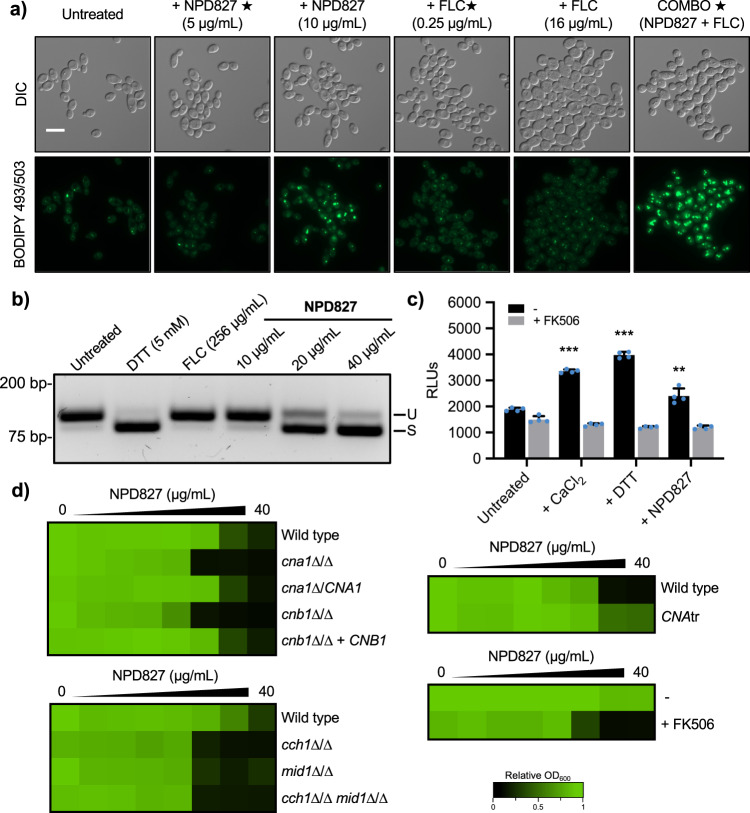
NPD827 induces diverse membrane-associated stress responses. **a** Lipid droplets were visualized by fluorescence microscopy of cells stained with BODIPY 493/503 after treatment with NPD827, fluconazole (FLC), or the combination (star indicates concentrations used in combination treatment). Experiment was repeated in three biologically independent replicates with similar results. Scale bar, 10 μm. **b** RT-PCR across the *HAC1* mRNA intron performed using total RNA extracted from *C. albicans* cells exposed to the indicated treatments. The size difference between the upper (U, unspliced) and lower (S, spliced) cDNA is 19 bp. Experiment was repeated in three biologically independent replicates with similar results. **c** A strain harboring a *UTR2p-lacZ* construct was incubated in YPD medium in the absence or presence of compounds as indicated. Relative calcineurin activation was monitored by measurement of β-galactosidase activity (RLU). FK506 was used to block activation of calcineurin under each inducing conditions. Data are expressed as mean ± SD of technical triplicates. *p*-value, compared to untreated: <0.0001 (CaCl_2_, DTT); 0.0024 (NPD827). *p*-value, compared to FK506: 0.0159 (CaCl_2_ + FK506); 0.0008 (DTT + FK506); 0.0007 (NPD827 + FK506). Significance was determined by one-way ANOVA with Bonferroni’s multiple comparisons test. Each treatment condition was compared to the wild-type untreated condition. *p*-value: **<0.005, ***<0.0005. Experiment was repeated in three biologically independent replicates with similar results. **d** Dose–response assays were performed in YPD medium, and growth was measured by absorbance at 600 nm after 24 h at 30 °C. Optical densities were averaged for duplicate measurements and normalized to no drug controls. Data are displayed as in Fig. 2b. *CNAtr* indicates a strain expressing a constitutively active allele of calcineurin^66^. Source data are provided as a Source Data file.

Lipid droplets are dynamic storage organelles that support homeostasis by sequestering excess free-lipid species, providing immediate protection from lipotoxicity, and serving as critical mediators of cellular stress responses^27^. Thus, we next investigated whether NPD827-induced changes to lipid composition altered the lipid-droplet content of cells. Treatment of *C. albicans* with NPD827 caused a concentration-dependent increase in both lipid-droplet number and size, as monitored by BODIPY 493/503 staining, compared to a vehicle-only control (Fig. 4a). Interestingly, treatment of *C. albicans* with either low (0.25 µg/mL) or high concentration (16 µg/mL) fluconazole did not increase lipid droplet content of cells; however, the combination of low-NPD827 with low-fluconazole greatly enhanced accumulation of lipid droplets (Fig. 4a).

The endoplasmic reticulum (ER) is the major site for synthesis of sterols and early sphingolipid intermediates^28^. This organelle relies on intrinsic stress responses, especially the unfolded protein response (UPR), to respond to the accumulation of misfolded proteins as well as disruptive changes in lipid composition^28^. Activation of the UPR occurs via auto-phosphorylation of the serine-threonine kinase and endoribonuclease, Ire1, resulting in alternative splicing of mRNA encoding the transcription factor *HAC1*^29^. Interestingly, lipid droplets have been implicated in the alleviation of ER stress^30^, sequestering excess free fatty acids and misfolded proteins^27,31^. To determine if NPD827 activates the UPR in *C. albicans*, a PCR-based assay was performed to monitor intron presence or absence in *HAC1* mRNA using cDNA prepared from NPD827-treated cells^32^. As observed with the ER stress-inducing positive control, DTT (5 mM), NPD827 drove splicing of *HAC1* message in a concentration-dependent manner, indicating that NPD827 activity induces ER stress (Fig. 4b). Notably, treatment with even a high concentration of fluconazole had no impact on *HAC1* splicing (Fig. 4b).

In addition to the formation of lipid droplets, ER stress triggers calcium influx through the Cch1-Mid1 calcium channel, leading to activation of stress responses regulated by the protein phosphatase calcineurin^33^. This calcium-calcineurin signaling pathway acts together with the UPR to promote long-term survival of cells undergoing ER stress^33^. Therefore, we examined whether NPD827 activates a calcineurin-dependent stress response using a well-established reporter system that exploits the downstream calcineurin effector, Crz1. The transcription factor Crz1 is activated by calcineurin in response to calcium, translocating to the nucleus, and up-regulating the expression of genes containing calcineurin-dependent response elements (CDREs) in their promoters. We used a strain harboring a construct with the CDRE-containing promoter *UTR2p* fused to *lacZ* to assess calcineurin activation in response to NPD827. The canonical calcineurin activator calcium chloride, as well as another ER stress-inducing compound DTT robustly activated this reporter as expected. NPD827 also activated reporter expression and the calcineurin inhibitor FK506 blocked this activation, confirming that transcriptional activation was indeed calcineurin-dependent (Fig. 4c).

Following up on these results, we examined whether modulating the activity of calcineurin in *C. albicans* would affect sensitivity to NPD827. Addition of the calcineurin inhibitor FK506, or deletion of either the catalytic (*CNA1*) or regulatory (*CNB1*) subunit of calcineurin, increased sensitivity to NPD827 (Fig. 4d). Additionally, a mutant lacking the auto-inhibitory domain of the phosphatase that causes constitutive activation of calcineurin (*CNAtr*), decreased susceptibility to NPD827 (Fig. 4d). To complement these calcineurin-related studies, we also investigated the effects of NPD827 on calcium signaling. In response to disturbances of Ca^2+^ homeostasis in the ER, Ca^2+^ influx through the plasma membrane is stimulated via a voltage-gated channel composed of Cch1 and Mid1^34,35^. Deletion of either *CCH1* or *MID1* resulted in a level of hypersensitivity to NPD827 similar to that seen in calcineurin-deletion mutants (Fig. 4d). Taken together, these results indicate that NPD827 disrupts lipid homeostasis in a far-reaching manner, which activates key stress-response pathways to cope with the imbalance.

### NPD827 rapidly disrupts membrane dynamics and impairs drug efflux

To begin investigating the molecular mechanism(s) by which NPD827 disrupts lipid homeostasis, we leveraged a chemical-genetic dataset in the model yeast *S. cerevisiae*^36^. The RIKEN NPDepo collection, including NPD827, has been profiled using a collection of mutants that encompass a diagnostic set of viable gene-deletions spanning all major biological processes^36^.This diagnostic resource can be used to make process-level target predictions by integrating the comprehensive genetic interaction data available in *S. cerevisiae*^36^. From such analysis, we uncovered several process-level Gene Ontology (GO) annotations based on interaction profile similarity, including, establishment of protein localization to the membrane (*p*-value = 0.00010), intracellular protein transport (*p*-value = 0.00013), posttranslational protein targeting to the membrane (*p*-value = 0.00018), intracellular protein transmembrane transport (*p*-value = 0.0002), and protein transmembrane transport (*p*-value = 0.0002)^36^.

**Fig. 5:**
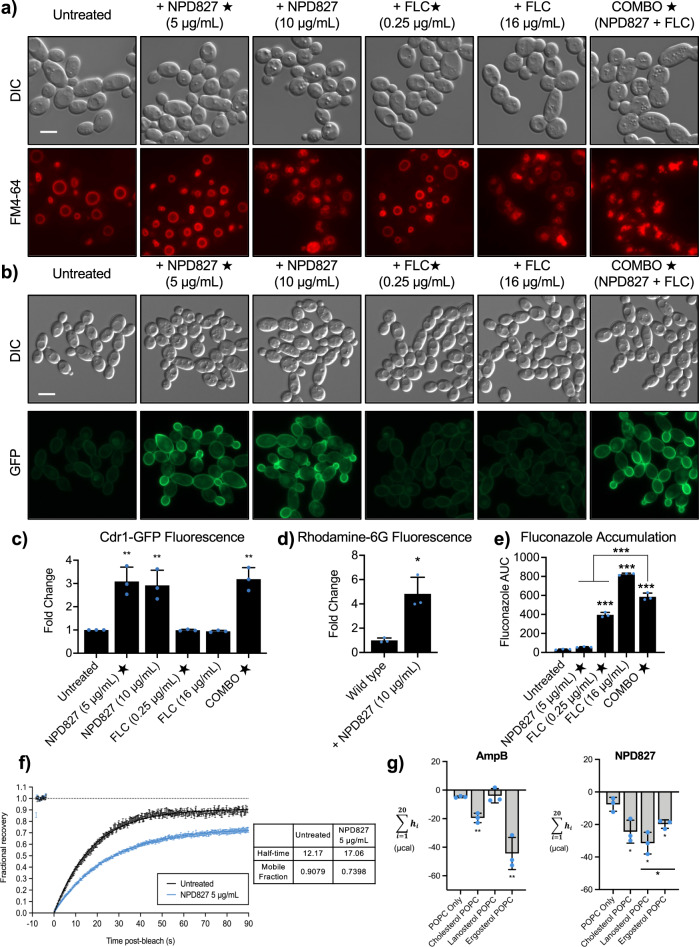
NPD827 alters membrane dynamics and perturbs multi-drug efflux. **a** FM4-64 staining of *C. albicans* (SN95) following treatment as indicated. Assays were performed in biological triplicate. Scale bar, 5 µm. **b** Fluorescent microscopy of Cdr1-GFP cells following treatment as indicated. Assays were performed in biological triplicate. Scale bar, 5 µm. **c** Quantification of cultures shown in **b** by flow cytometry. Bars represent median fluorescence intensity, normalized to untreated cells, as quantified by flow cytometry for at least 20,000 events. Data are expressed as mean ± SD for biological triplicates. *p*-value, compared to untreated: 0.0003 (NPD827 5 µg/mL); 0.0005 (NPD827 10 µg/mL); 0.0002 (Combination). Significance determined by by one-way ANOVA with Bonferroni’s multiple comparisons test, where all conditions were compared to wild-type, untreated control, *p*-value: **<0.01. **d** For Rhodamine-6G assays, *C. albicans* cells were treated with NPD827 (10 µg/mL) before staining with rhodamine-6G (1 µg/mL). Assays were performed in biological duplicate. Bars represent median fluorescence intensity, normalized to untreated cells, as quantified by flow cytometry for at least 20,000 events. Data are expressed as mean ± SD for biological triplicates. *p*-value: 0.0382 (NPD827 10 µg/mL). Significance determined by a two-tailed unpaired *t* test with Welch’s correction, *p*-value: *<0.05. **e** Relative intracellular concentrations of FLC were measured after treatment with NPD827 for 1 h. Data are presented as mean ± SD of technical triplicates (*n* = 3). Significance was determined by one-way ANOVA with Bonferroni’s multiple comparisons test, where all conditions were compared to wild-type, untreated control. *p*-value: ***<0.0005. Significance between the drug combo and FLC by one-way ANOVA, *p*-value <0.0001. *p*-value, compared to untreated: <0.0001 (FLC 0.25 µg/mL, 16 µg/mL, Combination). *p*-value, compared to NPD827 5 µg/mL: <0.0001 (Combination). *p*-value, compared to FLC 0.25 µg/mL: <0.0001 (Combination). **f** FRAP experiments with GFP-Ras1 *C. albicans*. GFP-Ras1 cells were incubated without or with 5 μg/mL NPD827 for the indicated duration before performing laser scanning confocal microscopy. Images were taken prior to and after photobleaching, and fluorescence recovery observed (10–90 s). Fluorescence recovery curves are shown for >25 cells per condition. **g** Total net isotherms from ITC interrogating amphotericin B (AmpB) and NPD827 binding to cholesterol, lanosterol, or ergosterol-containing, or sterol-free POPC LUVs. Data are presented as mean ± SD of technical triplicates. Significance was determined by unpaired *t*-test between each sterol-containing preparation and the sterol-free preparation and also for NPD827 between lanosterol- and ergosterol-containing conditions, *p*-value: *<0.05, **<0.005. *p*-value, compared to POPC only: AmpB, 0.0016 (Cholesterol); 0.0037 (Ergosterol); NPD827, 0.0243 (Cholesterol); 0.0137 (Ergosterol); 0.0065 (Lanosterol). *p*-value, compared to lanosterol: NPD827, 0.0471 (Ergosterol). Source data are provided as a Source Data file.

Given the broad representation of processes involved in protein trafficking, we investigated the effect of NPD827 on the endomembrane system and vacuolar morphology using a lipophilic dye, FM4-64. NPD827 treatment caused abnormal vacuolar morphology and the accumulation of endosome-like vesicles, suggesting impairment of trafficking by the compound, which was further exacerbated by a sub-inhibitory concentration of fluconazole (Fig. 5a). Previous studies in *S. cerevisiae* have implicated endocytosis and vacuole-mediated degradation in proper recycling of the ABC-transporter Pdr5, a homolog of *C. albicans* Cdr1^37^. Therefore, we examined the impact of NPD827-induced perturbation of the endomembrane system on localization of the multi-drug efflux pump, Cdr1. Using a GFP-tagged strain of Cdr1, we observed a dramatic increase in fluorescence at the plasma membrane upon treatment with NPD827, as qualitatively observed by fluorescence microscopy and quantified by flow cytometry (Fig. 5b, c and Supplementary Fig. 2a). This increase was not observed with fluconazole treatment alone, nor was the signal enhanced by drug combination (Fig. 5b, c). Importantly, despite increased Cdr1-GFP at the plasma membrane, NPD827 increased intracellular accumulation of a fluorescent efflux substrate, rhodamine-6G, indicating that drug efflux was actually impaired rather than enhanced (Fig. 5d). These NPD827-induced changes occurred very rapidly with Cdr1 accumulating at the membrane and efflux being impaired within 30 min, suggesting immediate rather than secondary effects of compound exposure. To confirm the hypothesis that NPD827-mediated impairment of Cdr1 function would lead to increased intracellular fluconazole accumulation, we measured fluconazole levels within the cell using LC–MS after 1 h of treatment. We detected significantly more intracellular fluconazole upon combination treatment (5 µg/mL NPD827, 0.25 µg/mL fluconazole) compared to the equivalent fluconazole concentration alone (0.25 µg/mL) (Fig. 5e). Notably, when measuring intracellular NPD827 levels, the addition of fluconazole did not lead to significantly increased NPD827 intracellular accumulation (Supplementary Fig. 2b), indicating that NPD827 treatment increases the bioavailability of fluconazole within the cell but not vice versa.

Given the rapid timescale on which NPD827 increased Cdr1 at the membrane and impaired efflux, we reasoned that NPD827 might directly impair membrane dynamics by altering the biophysical state of the plasma membrane, rather than inhibiting a specific protein target. To explore this possibility, we performed fluorescence recovery after photobleaching (FRAP) lateral mobility analysis using a *C. albicans* strain in which the GTPase Ras1 was GFP-tagged. We specifically used Ras1 as it is plasma membrane-associated, highly mobile, and lipid modifications are known to influence its dynamics within the membrane^38^. Upon treatment of this strain with NPD827 (5 µg/mL), the time to recover after post-photobleaching (i.e., half-time) was increased and a concordant decrease in the mobile fraction within the membrane was observed, both within minutes of compound addition (Fig. 5f). These observations suggest that NPD827 treatment causes measurable changes to the physical state of the plasma membrane, restricting mobility of at least some membrane-associated proteins.

To determine if the altered membrane dynamics seen by FRAP in whole cells could be explained by direct interaction of NPD827 with membranes, we moved to a cell-free, artificial system and used isothermal titration calorimetry (ITC) to monitor NPD827 interactions with lanosterol-, ergosterol-, or cholesterol-containing 1-palmitoyl-2-oleoyl-*sn*-glycero-3-phosphocholine (POPC) large unilamellar vesicles (LUVs)^39^. As previously shown, amphotericin B bound to both ergosterol- and cholesterol- but not lanosterol-containing LUVs (Fig. 5g). NPD827, on the other hand, bound to lanosterol-, ergosterol-, and cholesterol-containing LUVs (Fig. 5g). Interestingly, titration of NPD827 with lanosterol-containing LUVs yielded a significantly higher increase in total net exotherm compared to ergosterol-containing LUVs, suggesting that NPD827 may preferentially partition into membranous compartments where less mature intermediates within the sterol biosynthetic pathway reside. Consideration of both the whole-cell and biophysical findings acquired so far suggests that NPD827 disrupts lipid homeostasis by intercalating into sterol-containing membranes, thereby altering their biophysical properties, perturbing vesicular trafficking, impairing the function of membrane-associated proteins, and ultimately increasing intracellular accumulation of fluconazole.

### Mutations within the ESCRT-III complex confer resistance to NPD827 in a *C. albicans erg3∆/erg3∆* background

**Fig. 6:**
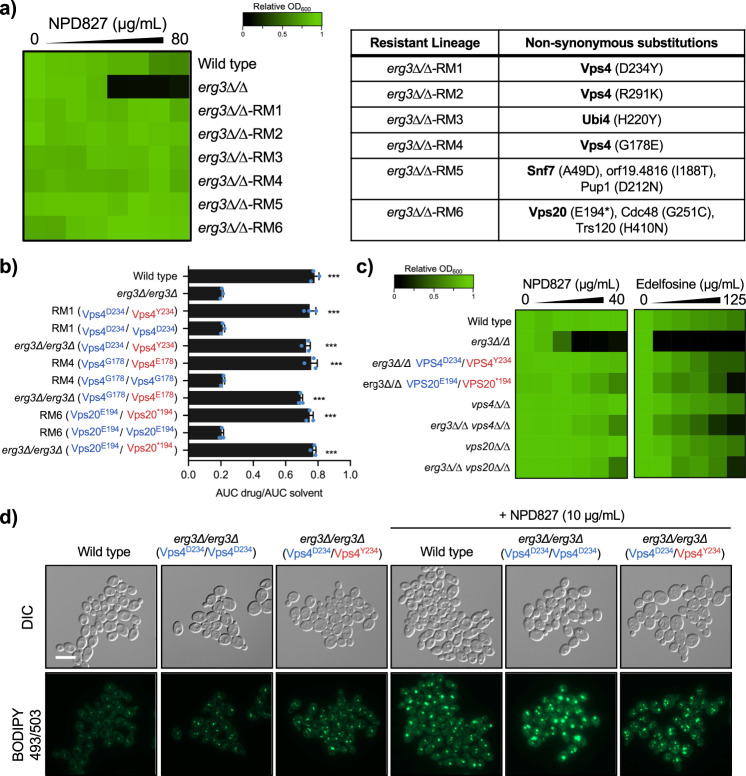
Selection experiments identify mutations in the ESCRT-III complex that confer resistance to NPD827 in an *erg3∆/erg3∆* background. **a** Single colonies from six independent lineages were tested for resistance to NPD827 and whole genome sequencing was used to identify candidate resistance mutations. Dose–response assays were performed in YPD medium, and growth was measured by absorbance at 600 nm after 24 h at 30 °C. Optical densities were averaged for duplicate measurements and normalized to no-compound, wild-type strain growth. Results are depicted as in Fig. 2b. Genome sequences were analyzed using Mutect (v1.1.7) and annotated using SnpEff (v4). Non-synonymous substitutions identified in each lineage are listed in the table. ESCRT-III complex members are bolded. **b** Allele-swap strains were assessed by growth curve analysis in the absence or presence of NPD827 (20 μg/mL) for 48 h in YPD at 30 °C, and growth was monitored every 15 min by absorbance at 600 nm. The areas under the resulting growth curves (AUC) were calculated and the ratio of AUC_drug_/AUC_solvent_ was normalized relative to a wild-type control. Data are expressed as mean ± SD of *n* = 3 biologically independent replicates. Significance determined by one-way ANOVA with Bonferroni’s multiple comparisons test, where all conditions were compared to the parent *(erg3∆/∆)*, ***<0.0005 *p*-value. *p*-value, compared to *erg3Δ/erg3Δ* parent: <0.0001 (Wildtype, RM1 [Vps4^D234/Y234^], *erg3Δ/erg3Δ* [Vps4^D234/Y234^], RM4 [Vps4^G178/E178^], *erg3Δ/erg3Δ* [Vps4^G178/E178^], RM6 [Vps20^E194/*194^], *erg3Δ/erg3Δ* [Vps20^E194/*194^]). **c** Dose–response assays were performed against allele-swap strains, *vps4∆/vps4∆*, and *vps20∆/vps20∆*, as previously described in (**a**). Alleles coloured in blue represent those found in the parent. Alleles coloured in red represent those found in the resistant lineage. Growth was measured using absorbance at 600 nm in YPD medium at 30 °C after 24 h. Measurements were normalized to no-drug controls and data are presented as in Fig. 2b. **d** Lipid droplets were visualized using fluorescence microscopy (GFP filter) of cells stained with BODIPY 493/503 after NPD827 treatment. Parental allele is displayed in blue, resistant-lineage allele is displayed in red. Experiment was repeated in three biologically independent replicates with similar results. Scale bar, 10 μm. Source data are provided as a Source Data file.

As an orthologous genetic approach to investigate how the sterol-interacting properties of NPD827 might confer azole-potentiating activity, we selected for NPD827-resistant isolates in a NPD827-sensitized *C. albicans* background that harbors a homozygous deletion of the ergosterol biosynthetic gene, *ERG3*. We selected for NPD827-resistant mutants by plating *erg3∆/erg3∆* cells (1 × 10^8^) on solid YPD medium containing NPD827 (40 µg/mL) and performed whole-genome sequencing of six independent resistant-isolates (Fig. 6a). Three of six resistant-mutants had non-synonymous heterozygous mutations in *VPS4*, a gene encoding an AAA-ATPase involved in transport from multi-vesicular bodies (MVBs) to the vacuole and in disassembly of the Endosomal Sorting Complex Required for Transport (ESCRT)-III complex. Two of six resistant-mutants had non-synonymous heterozygous mutations in genes encoding members of the ESCRT-III complex itself: *VPS20*, encoding a protein critical for initiation of ESCRT-III assembly, and *SNF7*, encoding the most abundant ESCRT-III subunit responsible for forming membrane-sculpting protein filaments. The remaining resistant isolate had a heterozygous non-synonymous mutation in *UBI4*, a gene encoding the ubiquitin precursor peptide required for tagging of proteins for degradation by the vacuole via the MVB pathway.

To verify the mutations identified by our sequencing analysis were necessary and sufficient for the resistant phenotype, we performed allele-swap experiments. The parental allele was introduced into the resistant mutant (RM) and reciprocally, the mutant allele was introduced into the parental strain. Expression of one allele of *VPS4*^D234Y^, *VPS4*^G178E^, or *VPS20*^E194*^ in an *erg3∆/∆* background conferred a level of resistance to NPD827 equivalent to *erg3∆/erg3∆*-RM1, -RM4, and -RM6, respectively, demonstrating that each of these mutant alleles is sufficient to confer NPD827 resistance (Fig. 6b). Reciprocally, replacement of the mutant allele with the ancestral *VPS4* or *VPS20* allele in the respective selected lineage abrogated resistance to NPD827, demonstrating that these mutations are necessary for resistance in these strains (Fig. 6b). Homozygous deletion of either *VPS4* or *VPS20* in the *erg3∆/erg3∆* background resulted in resistance to NPD827, suggesting that the identified mutations are not only loss-of-function but also dominant mutations (Fig. 6c). Interestingly, these mutations in *VPS4* or genes encoding members of the ESCRT-III complex were not necessary or sufficient for resistance in an alternative sensitized-background, *cna1∆/cna1∆*, and did not alter the potentiation of fluconazole activity by NPD827 in a wild-type background (Supplementary Fig. 3).

The NPD827-resistance conferring mutations identified were reminiscent of a resistance profile reported for the alkyl-lysophospholipid analog, edelfosine that is thought to bind to ergosterol in the plasma membrane resulting in disorganization of lipid microdomains and inducing internalization and vacuole-dependent degradation of membrane-bound proteins^40^. Loss-of-function of ESCRT-III has been proposed to provide an alternative route for membrane-bound enzymes and associated lipids to be recycled, which avoids their toxic accumulation within the vacuole. As with NPD827, we confirmed the heterozygous non-synonymous mutations identified in the NPD827-resistant lineages or homozygous deletion of *VPS4* or *VPS20* conferred edelfosine resistance in an *erg3∆/erg3∆* background (Fig. 6c). Loss-of-function of Vps4 or ESCRT-III restored the capacity of the cell to respond to lipotoxic stress. Increases in lipid-droplet size and vacuolar fragmentation caused by NPD827 were reduced compared to the *erg3∆/erg3∆* control (Fig. 6d and Supplementary Fig. 4a). However, these mutations did not reverse impairment of multi-drug efflux, as Cdr1-GFP rapidly accumulated at the plasma membrane and rhodamine-6G efflux was still impaired (Supplementary Fig. 4b). Taken together, the results support a mode of action for NPD827 that involves sterol-dependent targeting of membrane biophysics rather than inhibition of a specific protein target.

### NPD827 demonstrates therapeutic promise in preclinical models

**Fig. 7:**
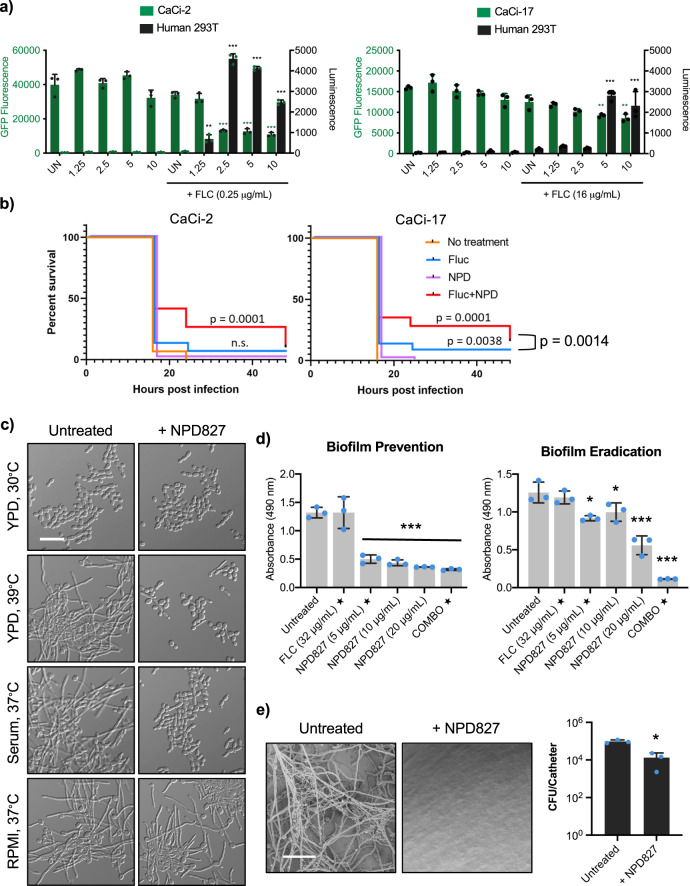
NPD827 reverses azole tolerance in co-culture and inhibits filamentation and biofilm development. **a** Co-culture of GFP-expressing *C. albicans* clinical strains with human 293T cells expressing a luciferase reporter. The effect on relative fungal (left axis, fluorescence) and human (right axis, luminescence) cell number by NPD827 in the presence or absence of fluconazole (FLC) after a 48-h treatment is depicted. Data are mean ± SD of technical triplicates. Significance was determined by one-way ANOVA with Bonferroni’s multiple comparisons test comparing each treatment condition to untreated (UN); *p*-value: *< 0.05, ** < 0.005, ***<0.0005. Data is representative of two independent biological replicates. *p*-value, compared to CaCi-2 + FLC 0.25 µg/mL: <0.0001 (NPD827 2.5, 5, 10 µg/mL). *p*-value, compared to 293T + FLC 0.25 µg/mL: 0.0023 (NPD827 1.25 µg/mL); <0.0001 (NPD827 2.5, 5, 10 µg/mL). *p*-value, compared to CaCi-17 + FLC 16 µg/mL: 0.0072 (NPD827 5 µg/mL); 0.0022 (NPD827 10 µg/mL). *p*-value, compared to 293T + FLC 16 µg/mL: <0.0001 (NPD827 5 µg/mL, 10 µg/mL). **b** *C. elegans* were infected with *C. albicans* clinical strains (CaCi-2, left panel; CaCi-17, right panel) and survival monitored in the presence of fluconazole, NPD827, the combination, or without treatment. 20 *C. elegans* worms were subjected to each condition, and survival was monitored over 48 h in three independent trials. Survival was plotted as a Kaplan–Meier survival curve. Log-rank (Mantel–Cox) test was used to determine *p* value. **c** Cultures were grown under filament-inducing conditions with the indicated compound treatment for 4 h. Scale bar, 10 μm. Images are representative of two independent biological replicates. **d** (Left) Compounds were added immediately to assess effects on biofilm formation. (Right) Compounds were added after 24 h of biofilm growth to assess effects on biofilm maturation. Metabolic activity was measured using an XTT reduction assay. Data are presented as mean ± SD of technical triplicates. Star indicates concentrations used in combination treatment. Significance determined by one-way ANOVA with Bonferroni’s multiple comparisons test, where all conditions were compared to wild-type untreated condition. *p*-value: *<0.05, **<0.005, ***<0.0005. *p*-value, compared to untreated: Biofilm Prevention, <0.0001 (NPD827 5, 10, 20 µg/mL, Combination); Biofilm Eradication, 0.0055 (NPD827 5 µg/mL); 0.0351 (NPD827 10 µg/mL), <0.0001 (NPD827 20 µg/mL, Combination). **e** *C. albicans* biofilms cultured in rat central venous catheters in the presence or absence of NPD827 (20 µg/mL) were examined by scanning electron microscopy at 24 h. Scale bar, 50 µm. Serial dilutions of catheter fluid were plated for fungal colony counts. Data represented as mean colony forming unit (CFU) per catheter ±SD of three technical replicates. Significance determined by a two-tailed unpaired *t*-test with Welch’s correction, *p*-value: *<0.05. *p*-value: 0.0071 (NPD827). Source data are provided as a Source Data file.

To begin evaluating the therapeutic potential of NPD827, we used in vitro co-culture models consisting of human 293T kidney-derived cells stably expressing firefly luciferase and clinical isolates of *C. albicans* (CaCi-2 and CaCi-17) expressing GFP (Fig. 7a). Assay plates previously seeded with 293 T cells were infected with *C. albicans* followed by addition of NPD827 in the absence or presence of a fixed concentration of fluconazole. After 48 h, relative fungal burden was measured by quantifying GFP fluorescence and relative rescue of human cell viability was evaluated by luminescence. NPD827 had minimal impact on fungal burden in the absence of fluconazole, but burden of both CaCi-2 and CaCi-17 was significantly reduced in the presence of sub-inhibitory concentrations of fluconazole (0.25 and 16 µg/mL, respectively), resulting in a reciprocal rescue of human cell survival, as measured by luciferase expression (Fig. 7a). While NPD827, alone or in the presence of fluconazole displayed minimal cytotoxicity to human cells in vitro (Figs. 1b and 7a), fluconazole does not inhibit biosynthesis of cholesterol, the analogous sterol in human cells to ergosterol in fungi. To ask whether NPD827 would increase the cytotoxicity of inhibiting cholesterol biosynthesis, we used lovastatin, an inhibitor of HMG-CoA reductase. This enzyme catalyzes an early, rate-limiting step in cholesterol biosynthesis. 293 T cells were cultured with a two-fold gradient of lovastatin in the absence and presence of NPD827. Combination with NPD827 has no impact on lovastatin cytotoxicity, highlighting the tolerability of NPD827 for human cells even when cholesterol synthesis is impaired (Supplementary Fig. 5).

As a non-optimized screen hit, NPD827 did not have the necessary pharmacological properties, especially adequate solubility, to permit testing in mouse models of systemic fungal infection. Therefore, we leveraged an alternative infection model involving the nematode *Caenorhabditis elegans*^41^. This simple invertebrate is frequently used as a model host to study *Candida*–host interactions due to ease of infection and functional similarity between the intestinal tract of *C. elegans* and mammals, a site from which *C. albicans* can invade to cause systemic infection. Worms were infected with CaCi-2 or CaCi-17 by allowing them to feed on plates previously inoculated with the fungal strains. After infection, they were transferred to multi-well plates containing solutions of the indicated compounds and their subsequent survival monitored for up to 48 h. Only combination treatment significantly extended the lifespan of worms infected with CaCi-2 (Fig. 7b). In worms infected with CaCi-17, a high concentration of fluconazole alone did significantly extend lifespan, but combination with NPD827 provided significant additional benefit (Fig. 7b).

To further explore the therapeutic potential of NPD827, we took an alternative approach to the worm model and determined whether NPD827-induced perturbation of membrane homeostasis would block a key virulence trait of *C. albicans*, the ability to transition between yeast and filamentous forms^42^. Disruption of lipid homeostasis, such as inhibition of ergosterol biosynthesis with fluconazole, has previously been reported to impair *C. albicans* filamentation in response to diverse cues^43^. We found that NPD827 alone blocked *C. albicans* filamentation induced by the host-relevant cues of serum and high temperature in rich YPD medium but was not sufficient to block filamentation induced by culture in RPMI medium at 37 °C (Fig. 7c).

Effects seen on filamentation encouraged us to evaluate activity against biofilms because the capacity to transition between yeast and filamentous forms is critical for robust biofilm development in *C. albicans*^44,45^. We performed in vitro assays in which *C. albicans* biofilms were established in static 96-well plates primed with calf serum under biofilm-inducing conditions (Fig. 7d). To examine effects of NPD827, cells were allowed to adhere to the polystyrene surface for two hours, washed to remove non-adherent cells, and cultured for 24 h in absence or presence of compound^46^. To test for effects on maturation, biofilms were prepared and allowed to establish for 24 h in the absence of drug, at which point NPD827 was added for an additional 24 h. In both experimental formats, biofilm burden was quantified by metabolic activity using an XTT reduction assay^47^. In both assay configurations, NPD827 significantly reduced biofilm metabolic activity, both in the absence and presence of fluconazole (Fig. 7d).

Finally, to evaluate the efficacy of NPD827 in a more clinically relevant context, we used a rat model of catheter-associated candidiasis that mimics a common complication of central venous catheters in patients^48^. Infection of implanted catheters with *C. albicans* was performed by intraluminal instillation of a fungal inoculum. Catheters were flushed after 6 h, instilled with either NPD827 (20 µg/mL) or control vehicle and then removed 24 h later. Serial dilutions of the catheter fluid were plated to assess viable colony-forming units (CFU), and catheters were processed for scanning electron microscopy (SEM). Under control conditions, biofilms became well-established in the indwelling catheters, as visualized by scanning electron microscopy (Fig. 7e). In contrast, treatment with NPD827 completely blocked the formation of *C. albicans* biofilms and reduced the recovered CFU by more than 7-fold compared to the untreated control (Fig. 7e). Together, these results provide proof-of-concept for the strategy of targeting lipid homeostasis with NPD827-like compounds to treat fungal infections.

## Discussion

**Fig. 8:**
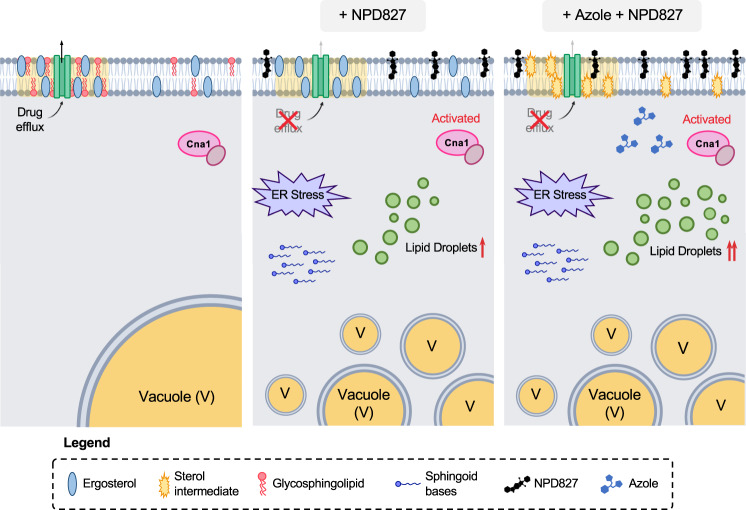
Model of NPD827 mechanism of action. (Left) Under normal physiological conditions, ergosterol and sphingolipids exist within the plasma membrane as lipid microdomains (yellow shadow). These microdomains harbor membrane-bound proteins, including drug-efflux pumps such as Cdr1. (Middle) NPD827 inserts into the plasma membrane and alters the biophysical properties of the membrane, reducing lateral mobility and impairing the function of membrane-bound proteins (i.e., Cdr1). Internalization of NPD827-associated membrane fragments disrupts the endomembrane system and induces vacuolar fragmentation, thereby perturbing lipid recycling throughout the cell. This disruption results in accumulation of toxic long-chain sphingoid bases, depletion of downstream sphingolipids, such as OH-Ceramide and Glucosylceramide, and induction of membrane-associated stress responses (i.e., lipid droplets, UPR, calcineurin-dependent stress responses). (Right) Due to impairment of drug-efflux by Cdr1, fluconazole accumulates within the cell further perturbing lipid homeostasis. The combination of effects ultimately halts growth of *C. albicans* in a synergistic manner.

With the striking rise in antifungal resistance worldwide, an urgent need exists for the development of alternative strategies to control drug-resistant fungal pathogens. Here, we identify a potent azole-potentiating agent with fungal-selective, broad-spectrum activity. We found that the imidazopyrazoindole NPD827 acts through a previously undescribed mode of action involving physical association with sterols (Fig. 8). This interaction alters the properties of fungal membranes, resulting in reduced membrane mobility, impaired function of some integral membrane proteins, and disruption of vesicular trafficking. As might be expected, the profound perturbation of membrane homeostasis caused by NPD827 induces a compendium of membrane-associated stress responses, including lipid-droplet accumulation, induction of the unfolded-protein response, and activation of calcineurin-dependent stress responses (Fig. 8). Combining NPD827 with fluconazole increases intracellular accumulation of fluconazole, which enhances disruption of sterol biosynthesis, increases lipid-droplet formation, and exacerbates vacuolar fragmentation (Fig. 8). Selection for NPD827-resistant mutants in an ergosterol biosynthesis-defective background yielded isolates with loss-of-function mutations in the multivesicular body pathway confirming that fungal membrane composition influences NPD827 activity and revealing that genetically altering vesicular trafficking can mitigate NPD827-induced lipotoxicity in this background.

Major success has been achieved in the development of drugs that target microbial cell membranes, including two out of the three major antifungal classes available for treatment of systemic fungal disease^2^. The azoles, which target ergosterol biosynthesis, have been effective in the clinic and agriculture, however, resistance has emerged as a major issue^2,3^. In contrast, the polyenes, which directly target the membrane by extracting ergosterol through a sterol sponge mode of action^23,49^, have proven to be exceptionally resistance-evasive for more than half a century. Moreover, when resistance does develop, it is associated with profound fitness costs including vulnerability to diverse exogenous stresses^39,50^. More broadly, the development of resistance to other membrane-targeting antibiotics also appears to occur less frequently than to agents with single protein targets^51–53^. Here, we show that NPD827 directly binds to sterol-containing lipid bilayers and induces membrane alterations within minutes that affect the function of membrane-bound proteins. Its effects on membrane homeostasis, however, are clearly distinct from that of the polyenes because NPD827 displays minimal single-agent activity. It is also notable that, in contrast to amphotericin B, NPD827 interacts strongly with lanosterol-containing lipid bilayers. The ability of NPD827 to interact with not just ergosterol, but also its precursor lanosterol, may explain why NPD827 activity is enhanced in combination with azole-mediated changes in membrane sterol composition. Finally, we show that NPD827 is capable of potentiating azoles in strains with well-characterized resistance mechanisms including overexpression of drug efflux pumps, substitutions in Erg11, and loss-of-function of Erg3^2^. This effect contrasts with other well-characterized molecules that enhance azole activity in *C. albicans* including inhibitors of the molecular chaperone Hsp90, the protein phosphatase calcineurin, and the protein kinase Pkc1^54,55^. While these stress response inhibitors enhance azole activity against azole-tolerant isolates and strains with mutations in *ERG3*, resistant isolates that overexpress efflux pumps remain resistant to the drug combination^54,55^.

Sterols and sphingolipids are distinct lipid classes that act together to maintain the integrity and function of eukaryotic membranes^56^. Genetic or pharmacological inhibition of the ergosterol biosynthesis pathway results in the depletion of ergosterol and the accumulation of specific sterol intermediates. Pathway inhibition, however, can impact more than just sterol levels. Some disruptions induce compensatory alterations in sphingolipid composition, suggesting that sterol and sphingolipid biosynthesis genes functionally interact^26,57,58^. Such interaction may contribute to the synergistic effects of combining NPD827, which affects both early and late sphingolipid intermediates, with fluconazole, which depletes ergosterol and drives accumulation of aberrant, toxic sterols. Beyond purely biochemical effects, the synergistic disruption of lipid homeostasis observed upon combining NPD827 and fluconazole may also occur because genes involved in sterol and sphingolipid biosynthesis are co-regulated by shared trans-acting factors^59^. For example, the transcription factors Ecm22 and Upc2 control ergosterol production, but they also have consensus binding sequences in the promoters of the SPT genes *LCB1* and *LCB2*^59^, and deletion of fatty acid elongases, involved in synthesizing sphingolipid precursors, induces *UPC2* expression in *C. albicans*^60^.

Sterols and sphingolipids are well-known to be connected in human diseases, especially Niemann Pick type C (NPC), where defects in sphingolipid and cholesterol trafficking appear to be interdependent^26,61^. Reminiscent of NPD827 treatment, mutations in *NPC1* or *NPC2*, which underlie the NPC disorder, cause accumulation of LCBs within the endocytic pathway and the increased production of lipid droplets^59^. Intriguingly, the AAA ATPase SKD1 (mammalian homolog of Vps4) physically interacts with NPC1 and regulates its role in endosomal cholesterol trafficking^62^. In yeast, *vps* mutants result in entrapment of Ncr1 (yeast homolog of NPC1) in pre-vacuolar compartments and homozygous deletion of *NCR1* renders cells resistant to the lipid analog, edelfosine^63,64^. Edelfosine directly binds to the N-terminal, lipid-binding tunnel of Ncr1 and this association is critical for its integration into the vacuolar membrane^64^. Given the structural similarity of NPD827 to sterols and the similar resistance profile it shared with edelfosine, NPD827 may interact with Ncr1 in a similar way. In this scenario, the NPD827 resistance-conferring loss-of-function mutations we identified within the ESCRT machinery may entrap Ncr1 in pre-vacuolar compartments and prevent NPD827 internalization via Ncr1-mediated shuttling to the vacuole, thus allowing lipids to be recycled back to the plasma membrane and ameliorating toxicity. Conversely, we found that the toxicity of NPD827-induced trafficking defects is exacerbated when alternate sterols are present in the plasma membrane, as with deletion of *ERG3*. This enhanced effect may be due to increased binding of NPD827 to alternative sterols within the membrane or due to increased cellular stress when trafficking defects occur in the absence of the mature membrane sterol, ergosterol.

Disruptions in lipid homeostasis are tightly linked with induction of key cellular stress responses that help mitigate lipotoxicity and promote cellular survival. Sphingolipids have emerged as important regulators of ER stress. As an example, addition of sphingolipid building blocks including palmitate, or the disruption of sphingolipid biosynthesis by genetic deletion of *ORM1/2*, results in activation of the UPR^65^. Further, lipid droplets act to buffer lipid uptake, distribution, and storage to finely tune lipid dynamics within the cell^27^. Pharmacological inducers of ER stress, including tunicamycin, brefeldin A, and thapsigargin, promote lipid droplet biogenesis in both yeast and mammalian cells^27^. Under conditions of ER stress, calcium- and calcineurin-dependent stress responses are also essential to sustain the UPR^33^. In particular, disruption of calcium influx via the Cch1-Mid1 Ca^2+^ channel or blocking downstream signaling by the calcium-dependent phosphatase calcineurin results in rapid cell death in response to ER stress^33^. Here, we demonstrate that these membrane-associated stress responses are activated in response to NPD827-induced lipotoxicity, and that deletion of components involved in the calcineurin-dependent stress responses results in hypersensitivity to NPD827. Of note, calcineurin-dependent stress responses also play an important role in the response of diverse fungal pathogens to azole-induced stress^66^. Further, a downstream effector of the calcineurin pathway, *RTA2*, is involved in calcineurin-mediated azole resistance and long chain sphingoid base release in *C. albicans*^67^. Deletion of *RTA2* results in exacerbation of azole-induced stress and the accumulation of dihydrosphingosine, similar to the effects of NPD827 on sphingolipid homeostasis^67^.

From a therapeutic perspective, NPD827 reveals an intriguing way to reverse azole resistance in *C. albicans* and inhibit key virulence traits, including filamentation and biofilm formation. Although NPD827 does not have the necessary pharmacological properties for systemic use, we demonstrated that NPD827 in combination with fluconazole does act in a fungal-selective manner in a co-culture model with human cells and in a *C. elegans* model to improve survival in the face of infection with azole-tolerant and -resistant clinical isolates. Contributing to its overall antifungal potential, NPD827 also impaired the key *C. albicans* virulence traits of filamentation and biofilm formation.While these results are encouraging from a therapeutic perspective, more work will be needed at both the basic science and drug-development levels to translate the new biological insights provided by discovery of NPD827 into a useful strategy to counter antifungal drug-resistance.

## Methods

### Strains and culture conditions

All strains used in this study are listed in Supplementary Table 1. All plasmids used in this study are included in Supplementary Table 2. All oligonucleotide sequences used in this study are included in Supplementary Table 3. All strains were cultured in YPD (1% yeast extract, 2% bactopeptone, and 2% d-glucose) for liquid culture growth, or YPD with 2% agar for solid medium growth, at 30 °C unless otherwise specified. Strains stocks were maintained at −80 °C in 25% glycerol.

### High-throughput chemical screen

A set of 19,840 compounds from the RIKEN Natural Product Depository (NPDepo) library were used to identify compounds that enhanced fluconazole activity against *C. albicans*. The NPDepo library is maintained as 1 mg/mL stocks in DMSO (dimethyl sulfoxide) and was screened at a final concentration of 10 µg/mL. A BioTech ADS-384-8 compound dispenser was used to transfer 0.2 µL of 1 mg/mL compound stocks in DMSO from each mother plate into four 384-well daughter plates. YPD medium alone or containing 1 µg/mL fluconazole was inoculated with ~10^3^ cells/mL of *C. albicans* (CaSS1) from a saturated overnight culture. Inoculum was aliquoted into 384-well microtitre plates (Greiner Bio-One #781182) to a final volume of 40 µL/well using a Ranin Liquidator^TM^ 96. Plates were incubated at 30 °C under static conditions in the dark, and growth was measured by absorbance at 600 nm after 24 h using a spectrophotometer (Molecular Devices SpectraMax M2). Percent growth inhibition was calculated relative to the average growth in DMSO control wells on each corresponding plate. Percent fluconazole potentiation was calculated by comparing the growth in the presence of fluconazole compared to no fluconazole controls for each individual compound. Azole potentiator screen hits were defined as significantly inhibiting growth inhibition in the presence of fluconazole and significantly potentiating fluconazole compared to no fluconazole controls (cut-off: 20 × mean absolute deviations [MAD] from the median). Hits were then validated in a secondary screen under the same conditions and compounds that displayed reproducible activity were then re-ordered from an independent supplier and used for all subsequent analyses.

### Dose–response, checkerboard, and growth kinetic assays

Antifungal susceptibility was measured in flat bottom, 384-well microtitre plates (Greiner Bio-One #781182) using a dose–response protocol described in ref. ^68^. In brief, dose–response assays were set up in 2-fold serial dilutions of either NPD827 (InterBioScreen, STOCK1N-26843), doxycycline (Bio Basic Canada), or edelfosine (Sigma-Aldrich, SML0332) in a final volume of 40 µL. Checkerboard assays were performed similarly to the dose–response assays; two-fold serial dilutions of NPD827 along the *y*-axis, and two-fold serial dilutions of either fluconazole (Sequoia Research Products Ltd.), voriconazole (Sequoia Research Products Ltd.), caspofungin (generously provided by Terry Roemer from Merck Research Laboratories), terbinafine (Sigma-Aldrich), fenpropimorph (Riedel-de Haen), amphotericin B (Sigma-Aldrich), and myriocin (Cayman Chemicals) along the *x*-axis. All drug stocks were prepared in DMSO except for fluconazole, caspofungin, and edelfosine, which were prepared in sterile ddH_2_O. Cell densities of overnight cultures were determined, and dilutions were prepared such that ~10^3^ cells were inoculated into each well. Plates were incubated in the dark at 30 °C for 24–48 h as indicated, at which point the absorbance was determined at 600 nm using a spectrophotometer (Molecular Devices) and corrected for background from corresponding medium. Growth was normalized to the no-drug treatment well for the relevant strain. Plotted are the average optical density values of technical duplicate measurements. Each strain was tested in at least two biological replicates. Dose–response and checkerboard assay data was quantitatively displayed with color using the program Java TreeView 1.1.3 (http://jtreeview.sourceforge.net).

For checkerboards against *A. fumigatus*, frozen glycerol stocks were used to inoculate a PDA (2.4% potato dextrose broth and 2% agar [w/v]) plate and cells were incubated at 37 °C for 48 h or until robust conidiation was observed. This master plate was used to inoculate an additional PDA plate, which was then incubated again at 37 °C for 48 h or until robust conidiation was observed. To harvest conidia, 10 mL of Tween saline solution (0.025% [v/v] Tween80, 0.8% NaCl [w/v]) was poured onto the PDA agar plate and a rubber spatula was used to scrape the surface. Conidia solution was then filtered through four layers of Miracloth into a 15 mL falcon tube. Conidia were counted using a haemocytometer and diluted to a final concentration of 2.5 × 10^4^ conidia/mL in YPD. Diluted inoculum was aliquoted into 96-well microtitre plates (Sarstedt # 83.3924) containing drug dilutions to a final volume of 200 µL. Plates were incubated in the dark at 37 °C for 48 h, at which point 20 µL of 0.03% (w/v) resazurin salt solution was added for 4 h. Fluorescence was measured on a plate reader (Tecan Infinite M1000Pro, 480 nm excitation and 540 nm emission) and wells were then imaged. Fluorescence signal was normalized to the no-drug well and optical density values of technical duplicate measurements were averaged. Checkerboard assay data was quantitatively displayed with color using the program Java TreeView 1.1.3 (http://jtreeview.sourceforge.net). Experiments were performed in biological duplicate, with one representative shown.

For growth kinetic assays, cultures were grown overnight in YPD, diluted to an OD_600_ of 0.1 in 40 µL of YPD without or with NPD827 in 384-well plates, and grown at 30 °C with continuous shaking (TECAN GENios). OD_595_ was measured every 15 min for 48 h with XFluor4 software. All cultures were grown in technical triplicates. Growth curves were generated for at least two biological replicates. Data was plotted using GraphPad Prism.

### Extraction and mass spectrometry analysis of sterols and sphingolipids

For sterol profiling, overnight cultures were pelleted at 2095×*g* for 5 min and washed once with 10 ml synthetic complete without ammonium sulfate + 2% glucose (SC) medium once. Cultures were diluted to the final OD_600_ of 0.4 in 50 ml SC medium + DMSO (equal volume), and respective compounds and incubated for 6 h at 30 °C. Cells were pelleted at 2095×*g* for 5 min at 4 °C and washed once with ice-cold phosphate buffered saline. Pellets were weighted and flash frozen with liquid nitrogen and stored at −80 °C.

Total intracellular sterols of yeast were extracted as reported previously with slight modification^69^. Cell pellets were mixed with 3 ml extraction solution (25% KOH w/v, 64% methanol v/v) adding 30 µl of 2 mg/ml cholesterol (dissolved in chloroform) as an internal standard in borosilicate glass tubes and incubated at 80 °C water bath for 1 h and vortexed every 20 min. After incubation, the samples were cooled to room temperature and 1 ml water and 3 ml chloroform were added to each sample for extraction. The tubes were vortexed for 10 s 3 times then centrifuged at 1048×*g* for 5 min. Using Pasteur pipettes, the bottom organic phases were transferred to another borosilicate glass tubes and dried by Genevac (EZ-2 Series SP Scientific) on the high boiling point setting. Samples were dissolved in 600 µl chloroform and 200 µl was transferred into a 1 ml conical thick-walled glass reaction vial for derivatization^70^. Saponified lipid extracts were evaporated to dryness under a stream of N_2_ gas at an airflow rate of 1 mL/min and the bottom of the tubes suspended in a 40 °C-water bath. Dissolved the dried down extract in 20 µL pyridine and 30 µL MSTFA by flicking bottom of the vial. Then the lipid extract was incubated in a dry heating block at 37 °C for 30 min to derivatize. 20 µL derivatized lipid extract was removed into a GC auto-sampler vial, 1 µL extract was then injected into the GC–MS for analysis. Samples were injected at 200 °C into a 30 m 122–5532 Agilent DB-5MS polysiloxane column (0.25 mm i.d.) coated with 50% methyl and 50% phenyl groups coupled to an intermediate polarity fused silica guard column 5 m in length with a 0.53 mm diameter. Helium was the carrier gas at a flow rate of 1 ml/min. The oven temperature was isocratic at 50 °C for 2.5 min and increased to 70 °C at a rate of 7.5 °C/min, then the temperature was programmed to 310 °C at a rate of 5 °C/min and held for 1 min. The oven was then cooled down to 50 °C before injection of the next sample. The MS detector was turned on at 6 min and the mass spectra was recorded at 0.9 scans/s with a scanning range of 50–700*m*/*z*. The data were collected in SIM mode.5. Peak areas of sterols were normalized to that of internal extraction efficiency control (cholesterol) and the wet weights of each sample. All assays were done in triplicate and are representative of biological duplicates. Data are plotted in Prism v9.3.0 (GraphPad) showing the mean relative quantity ±SD.

For sphingolipid profiling, saturated overnight cultures of SN95 were diluted to an OD_600_ of 0.2 in 10 mL of minimal medium (0.17% yeast nitrogen base without amino acids without ammonium sulfate, 2% d-glucose, 0.1% monosodium glutamate, 1X histidine, 1X arginine) without drug treatment. Cells were grown for 2 h before adding drug and incubating for an additional 4–5 h with shaking at 30 °C. Samples were normalized to ~5 × 10^8^ cells/replicate and then transferred into reusable glass tubes with phenolic screw caps. Samples were pelleted by centrifugation and washed with 1X PBS before flash freezing with liquid nitrogen. Internal standards (C17-ceramide and C17-sphingosine) were added to the samples, and sphingolipids were extracted as described previously^71^. Briefly, fungal cells were suspended in Mandala buffer (ethanol:distilled water:diethyl ether: pyridine:ammonium hydroxide 15:15:5:1:0.018; v/v) and submitted to 2 cycles of vigorous vortexing for 20 s, sonication for 20 s and incubation at 60 °C for 15 min^72^. The lipid extract obtained was dried and solubilized in methanol: chloroform (2:1, v/v), followed by the addition of 1/3rd volume of chloroform and 1/3rd volume of distilled water^73^. The organic phase was collected and 1/3rd of each sample was reserved for inorganic phosphate (Pi) determination to normalize the lipid amounts^71^. The remaining 2/3rd of each sample was submitted to a mild alkaline methanolysis of phospholipids^74^. The organic phase was transferred to a new tube, dried and used for LC–MS analysis, which was performed in a Thermo Finnigan TSQ Quantum Ultra Mass Spectrometer (Thermo Fischer Scientific, USA).

### Fluorescence microscopy

Stationary phase *C. albicans* cultures were diluted to an OD_600_ of 0.2 and grown at 30 °C for 2 h, shaking, in the absence of drug. After 2 h, NPD827 (or DMSO equivalent) was added to corresponding tubes and cultures were grown at 30 °C shaking for 1 h. Cells were centrifuged, media was removed, and cells were washed once in 1X PBS. To visualize lipid droplets, cells were stained with 1 μg/mL BODIPY (Cayman Chemical, 25892) for 10 min at 30 °C and washed twice in 1X PBS before microscopy. Lipid droplets were visualized using fluorescence microscopy using a GFP filter. To visualize drug efflux, cells were stained with 1 μg/mL Rhodamine-6G (in sterile ddH_2_O; Sigma-Aldrich) for 30 min at 30 °C and washed twice in 1X PBS before microscopy. To visualize FM4-64-stained cells, cells were stained with 8 µM 1 μg/mL in the dark for 30 min at 30 °C. After 30 min, cells were resuspended in 1 mL YPD medium to remove free FM4-64 (Thermo Fisher Scientific, #T3166) and added to 4 mL of fresh YPD medium. Cells were incubated in FM4-64 free medium for 90 min at 30 °C, spun down, and resuspended in 1X PBS. Rhodamine-6G and FM4-64 were visualized using fluorescence microscopy using an RFP filter. To quantify fluorescence, a CytoFlex Flow Cytometer (Beckman Coulter) was used. Cells were added to a flat bottom, transparent, 96-well plate (Beckman Coulter). Each sample was run using the CytExpert Software (version 2.4) until ~20,000 events had been recorded. Populations were gated to exclude debris and doublets, and the median value was taken for each sample.

For Cdr1-GFP experiments, CaLC7116 was subcultured in YPD medium in 3 mL at an OD_600_ 0.2 for 3 h with agitation. Indicated compounds were added and cells were incubated for an additional 30 min. Cells were then pelleted by centrifugation at 3000×*g* for 5 min and washed 2× in PBS. Samples were then imaged by microscopy or flow cytometry as indicated in the “Methods” section currently. All assays were done in biological triplicates shown in the figure. Data are plotted in Prism v9.3.0 (GraphPad) showing the mean fluorescence ±SD.

### *HAC1* mRNA splicing assay

To test for the activation and alternative splicing of *HAC1*, reverse transcription-polymerase chain reaction (RT-PCR) was performed on RNA extracted from *C. albicans*, as previously described^32^. Stationary phase SN95 cells were used to inoculate a fresh culture at an OD_600_ of 0.2 and grown to mid-exponential phase (OD_600_ = 0.6) in YPD at 30 °C with shaking. Cells were then exposed to 5 mM dithiothreitol (DTT), 10 μM tunicamycin, or varying concentrations of NPD827 for 1 h. Control cultures were treated with the equivalent volumes of DMSO. RNA was isolated using QIAGEN RNeasy kit and further treated with RNase-free DNase (QIAGEN). cDNA was prepared using the AffinityScript cDNA synthesis kit. The reaction products (2 μL) were then mixed with 1 μL each of 10 μM *HAC1* primers (oLC4345/oLC4347), 2 μL of 2.5 mM each dNTP mix, 2.5 μL of the supplied 10X PCR buffer, 16.37 μL of ddH_2_O, and 0.13 μL of TAKARA Taq DNA polymerase (5 U/μL), and subjected to a 30-cycle thermal cycle reaction of 94 °C for 30 s, 54 °C for 30 s, and 72 °C for 60 s. PCR products were run on 2% agarose gels (1X TAE Buffer) at 100 V for 60 min and the SYBR Safe stained fluorescent images were captured by a ChemiDoc.

### Calcineurin activation assay

For β-Galactosidase assays with the *UTR2p-lacZ* reporter, stationary phase *C. albicans* cultures were diluted to OD_600_ of 0.5 and grown at 30 °C for 2 h, shaking. After 2 h, 100 μL of culture was placed into a 96-well plate (Sarstedt) with test compounds and the plate was grown at 30 °C shaking for another 4 h. Following this, cell cultures were diluted 1:100 in YPD and 30 μL of diluted culture was placed into a 384-well white assay plate (Corning). Pre-diluted Gal-Screen substrate diluted 1:25 with Gal-Screen Buffer was added in equal volume to the 30 μL of diluted culture, and the assay plate was placed in TECAN to read luminescence every 15 min for 1 h. Data were normalized to OD_600_ values read with a spectrophotometer after 4 h of compound treatment. All treatments were performed in triplicate and are representative of biological duplicates.

### Intracellular compound quantification

To quantify intracellular fluconazole levels, *C. albicans* (SN95) was subcultured from overnight cultures at a starting OD_600_ of 0.4 in 5 mL of YPD in the presence of the indicated compound concentration for 1 h with agitation. Cells were then transferred to falcon tubes and pelleted at 3000×*g* for 5 min at 4 °C. Media was removed, and cells were washed with 5 mL of cold PBS 3 times with centrifugation of 2000×*g* for 5 min in between. Cells were resuspended in 1 mL cold PBS, flash frozen in liquid nitrogen, and stored at −80 °C overnight. The following day, cells were thawed on ice, 25 µL of 6 N NaOH was added to each falcon tube, and samples were vortexed for 15 s. 500 µL of 10 mM sodium phosphate (pH 6.0) was added to each sample followed by vortexing for 15 s. Compounds were extracted with 5 mL of CH_2_Cl_2_ and vortexed for 5 min, followed by centrifugation for 10 min at 4000×*g* at 4 °C. The organic phase was collected and dried. Before subsequent LC–MS analysis, samples were resuspended in 75 µL of 1:1 acetonitrile:water (MeCN:H_2_O). The resuspended cell extracts were injected (10 µL) in triplicate on to an Acquity UPLC (BEH C18 column (1.7 μm, 2.1 × 50 mm)) I-Class coupled to a Xevo G2-S QToF (Waters). Gradient was 10% B to 95% B over 8 min at 0.125 mL min^−1^ (A: water + 0.1% (v/v) formic acid, B: acetonitrile + 0.1% (v/v) formic acid). Ionization occurred with an electrospray ionization (ESI) source in positive ion mode. Fluconazole was detected and quantified using selected reaction monitoring mass transition 307.110 [M + H] + → 220.0685 using TargetLynx for peak finding, smoothing, and area quantitation. All samples were run in biological and technical triplicate and a representative replicate was plotted in GraphPad Prism.

To quantify intracellular NPD827 levels, CaLC239 (SN95) was subcultured from a saturated overnight culture at an OD_600_ of 0.4 in 5 mL of YPD in the presence of the indicated compound concentration for 1 h with agitation. Cells were then transferred to falcon tubes and pelleted at 3000×*g* for 5 min at 4 °C. cells were washed with 5 mL of cold PBS 3 times at 2000×*g* for 5 min, flash frozen in 1 mL PBS and stored in −80 °C overnight. Pellets were thawed on ice and 3 μL of 10 μg/mL verapamil was spiked in. 25 μL of 6 N NaOH was added and samples were vortexed for 15 s, 500 μL of 10 mM sodium phosphate pH 6 was added and sampled were vortexed for 15 s. 5 mL of CH_2_Cl_2_ was added to the tubes and samples were vortexed for 5 min and subsequently centrifuge for 10 min at 4000×*g* at 4 °C. The bottom organic phase was collected and dried by Genevac (EZ-2 Series SP Scientific) on the very low boiling point setting. 200 μl DMSO was added and 10 μL sample was analyzed by HR-ESI-MS (Agilent 1290 Infinity II Series HPLC system coupled to qTOF 6550 ESI/MS) operated in positive ion mode with mobile phase A (0.1% formic acid in water) and mobile phase B (0.1% formic acid in acetonitrile) at a flow rate of 0.4 ml/min using Eclipse XDP C8 column(2.1 × 100 mm, 3.5 µm). Peak areas of compounds were normalized to that of internal verapamil standards to control for extraction efficiency and optical density. All assays were done in technical triplicate and biological duplicates. Data from both biological duplates are plotted in Prism v9.3.0 (GraphPad) showing the mean relative quantity ±SD.

### FRAP analysis

FRAP experiments were conducted on an A1R point-scanning confocal system (Nikon). A saturated overnight culture of *C. albicans* strain (CaLC7464), which expresses GFP-Ras1 at the plasma membrane, was subcultured 1:100 into fresh YPD. After 2 h, growth at 30 °C with agitation, 5 × 10^5^ cells were plated on 18 mm glass coverslips that had been previously treated with 2 mg/mL concanavalin A. Cells were allowed to settle by gravity for 15 min, and places in 1 × PBS containing 2% glucose for imaging at 30 °C. Prior to imaging, *C. albicans* was treated with 5 µg/mL NPD827 for 20 min at 30 °C. Images were acquired using a ×100/1.4 NA oil objective (Nikon), 1.2-AU pinhole, Galvano scanning mode, with no line averaging, at 256-pixel resolution and with 1.83 × rectangular zoom. FRAP acquisition was at 3.7 fps, using the 488-laser line at ~1% power. For a single field, after 5 s of initial imaging, 3 regions of interest ~1.5 µm in diameter was bleached sequentially for 1 s each using the 405 laser at 100% power, followed by imaging 2 min for fluorescence recovery. Images were exported and analyzed for fluorescence intensity using Volocity software. After background subtraction, fluorescence intensity units were normalized to a non-bleached portion of the membrane and transformed to a 0–1 scale to correct for differences in bleaching depth and allow for comparison of up to 30 individual FRAP curves per condition^75^. Normalizations were done using Microsoft Excel software (v16.60). GraphPad Prism software (v9.2.0) was used to fit the FRAP curves to a single exponential, plotted as fractional recovery over time. Values derived from this non-linear regression were used to calculate mobile fraction, half-time of recovery, and diffusion rate^76^.

### Isothermal titration calorimetry

Experiments were performed using a NanoITC isothermal titration calorimeter (TA Instruments). Solutions of the compounds to be tested were prepared by diluting a 15 mM DMSO stock solution to 150 µM with K buffer (5 mM HEPES/KHEPES, pH = 7.4). LUV solutions were diluted with buffer and DMSO to give a final phospholipid concentration of 8 mM in a 1% DMSO/K buffer solution. Immediately prior to use, all solutions were degassed under vacuum at 25 °C for 10 min. To prepare LUVs, a 13 × 100 mm test tube was charged with 800 µL of palmitoyl oleoyl phosphatidylcholine (POPC in CHCl_3_, Avanti Polar Lipids) and 230 µL of sterol solution. For sterol-free LUVs, a 13 × 100 mm test tube was only charged with POPC. The solution was removed, and the resulting lipid film was stored under high vacuum for a minimum of 8 h prior to use. The film was hydrated with 1 mL of K buffer and vortexed vigorously for 3 min to form suspension of multilamellar vesicles (MLVs). The resulting lipid suspension was pulled into a Hamilton 1 mL gastight syringe and was placed in an Avanti Polar Lipids Mini-Extruder. The lipid solution was then passed through a 0.20 µm Millipore polycarbonate filter 21 times to prevent carry over of MLVs into the LUV solution. Titrations were performed by injecting the LUV suspension at ambient temperature into the sample cell (volume = 0.191 mL), which contained the compound of interest. Consistent with standard procedure^77^, the volume of the first injection 0.23 µL (not included in final analysis) and next, nineteen 2.50 µL injections of the LUV suspension was performed with 300 s between each injection. The rate of stirring for each experiment was 350 rpm. NanoAnalyze software (TA Instruments) was used for baseline determination and integration of the injection heats prior to subtraction of dilution heats and calculation of overall heat involved. The overall heat involved during the experiment was calculated using the following formula (1):

$${{{{{{\rm{\mu cal}}}}}}}_{{{\rm {overall}}}}=\mathop{\sum }\limits_{i=1}^{n}(\Delta {h}_{{{\rm {injection}}}}^{i}-\Delta {h}_{{{\rm {injection}}}}^{n})$$

where *i* is the injection number, *n* is the total number of injections, $\Delta {h}_{{{\rm {injection}}}}^{i}$ is the heat of the *i*th injection $\Delta {h}_{{{\rm {injection}}}}^{n}$ is the heat of the final injection of the experiment. All experiments were performed in triplicate and mean ± standard deviation was plotted in GraphPad Prism.

### Selection experiments and whole genome sequencing

To select for NPD827-resistant mutants, overnight cultures were grown in YPD medium at 30 °C and cell densities were quantified by haemocytometer. Cells (~1 × 10^8^) were plated on YPD containing 40 μg/mL of NPD827. As a control, ~1 × 10^8^ cells were plated on YPD containing DMSO. Selection plates were incubated at 30 °C for 2–3 days, single colonies were isolated, and subsequently tested for resistance phenotypes. These selection experiments were performed in six independent biological lineages. Overnight cultures of pure resistant mutant colonies were used to isolate genomic DNA for whole genome sequencing. DNA was prepared using the PureLink Genomic DNA Mini Kit (Invitrogen) according to the manufacturer’s instructions. Sequencing libraries were prepared using the Nextera XT Kit (Illumina) according to the manufacturer’s instructions. Libraries were sequenced on the Illumina NextSeq platform using paired end reads (300 bp). Sequence reads were de-multiplexed and trimmed to remove bases with Phred scores <Q30. Reads were aligned to the SC5314 reference genome from CGD using Bowtie2 (v2.4.0)^78^, and the alignment was visualized using Integrative Genomics Viewer^79^. MuTect (v1.1.7) was used to identify unique mutations compared to the parental strain^80^.

### Filamentation microscopy

The filament-inducing cues tested include YPD medium at the elevated temperature of 39 °C, YPD medium containing 10% (vol/vol) heat-inactivated newborn calf serum at 37 °C, and RPMI medium, pH 7.0 at 37 °C. Overnight cultures of *C. albicans* (SN95) were diluted to an OD_600_ of 0.2 and grown under the conditions specified while shaking for 4 h. All imaging was performed using DIC microscopy on Zeiss Axio Imager.MI at ×100 magnification.

### In vitro biofilm drug susceptibility

Drug susceptibility assays were performed on biofilms as previously described^81^. Briefly, strains were grown overnight in YPD at 30 °C. Subsequently, cultures were resuspended in RPMI medium buffered with MOPS to a final concentration of 10^6^ cells/mL. An aliquot of 100 μL was added to each well of a 96-well flat-bottom plate, followed by incubation at 37 °C. After 2 h, wells were gently washed twice with 1X phosphate-buffered saline (PBS) to remove non-adherent cells. To assay for biofilm prevention, fresh medium was added with or without NPD827 and/or fluconazole. After 24 h, non-adherent cells were washed away with PBS and biofilm cell metabolic activity was measured using the XTT reduction assay, as previously described^82^. Briefly, 90 μL of XTT at 0.5 mg/mL and 10 μL of phenazine methosulfate at 320 μg/mL were added to each well, followed by incubation at 37 °C for 15 min. Absorbance of the supernatant transferred to a fresh plate and diluted 1:4 in 1X PBS was measured at 490 nm using a spectrophotometer. To assay for biofilm eradication, fresh medium was added to adhered cells without any drug and incubated for 24 h to allow for establishment of mature biofilms. Non-adherent cells were then washed away with 1X PBS and fresh medium containing either NPD827 and/or fluconazole was added. After 24 h, non-adherent cells were washed away with 1X PBS and biofilm cell metabolic activity was measured using the XTT reduction assay, as previously described. Experiments were carried out in a minimum of three technical replicates for each strain/condition.

### In vivo biofilm drug susceptibility

In order to evaluate biofilm formation in vivo, a rat central venous catheter infection model was employed^48^. Rat catheter biofilm animal procedures were approved by the Institutional Animal Care and Use Committee at the University of Wisconsin-Madison according to the guidelines of the Animal Welfare Act, The Institute of Laboratory Animals Resources Guide for the Care and Use of Laboratory Animals, and Public Health Service Policy. The approved animal protocol number is DA0031. Spraw Daley (purchased from Envigo) male rats, 10 weeks old were used for the rat catheter experiments. In brief, a heparinized (100 U/mL) polyethylene catheter was surgically inserted into the jugular vein and advanced 2 cm to a site above the right atrium. After the catheter was secured to the vein, the proximal end was tunneled subcutaneously to the midscapular space and externalized through the skin. The catheters were implanted 24 h prior to inoculation with *C. albicans* to allow a conditioning period for deposition of host protein on the catheter surface. Infection was performed by intraluminal instillation of 500 µL of *C. albicans* (10^6^ cells/mL) with or without 20 µg/mL NPD827. After 6 h, the catheters were flushed and maintained with heparinized 0.85% NaCl with or without NPD827 for 24 h to allow biofilm growth. At the end of the observation period, the animals were sacrificed, and the catheters were removed. To image biofilm growth, catheters were fixed overnight in 4% formaldehyde and 1% glutaraldehyde in PBS. Catheters were then washed with PBS and treated with 1% osmium tetraoxide in PBS for 30 min. Alcohol washes were used to dry the segments before they were mounted, and gold coated. All images were taken using a scanning electron microscopy (JEOL JSM-6100) in the high-vacuum mode at 10 kV. To quantify fungal biofilm formation in the catheter, the contents were drained to remove blood and non-adherent organisms. The distal 2 cm of catheter was cut from the entire catheter length and the segment was placed in 1 mL of 0.85% NaCl. Following sonication for 10 min (FS 14 water bath sonicator and 40-kHz transducer [Fisher Scientific]) and vigorous vortexing for 30 s, serial dilutions of the catheter fluid were plated on sabouraud dextrose agar (SDA) for viable fungal colony counts. Results are expressed as the mean colony forming unit (CFU) per milliliter.

### Mammalian cell toxicity and co-culture experiments

To assess mammalian cell toxicity, HepG2 cells were grown to 70% confluence in DMEM (Sigma-Aldrich) supplemented with 10% heat-inactivated FBS (Gibco) in 5% CO_2_ at 37 °C. Cells were rinsed with cell-culture grade PBS without Ca^2+^ and Mg^2+^ (Sigma-Aldrich) and incubated with 1 mL 0.05% Trypsin–EDTA dissociation reagent (Gibco) for 5–10 min. Cell densities were quantified using a haemocytometer and diluted to 1 × 10^5^ cells/mL (2000 cells/well) in DMEM-FBS. 20 μL aliquots of the cell suspension was added to each well of a 384-well, flat clear bottom black microtiter plates (Corning) and incubated at 37 °C for 16 h. Broth microdilutions of compounds of interest were made in a new 96-well plate at 2X desired final concentration in DMEM-FBS. 20 μL aliquots of the 2X broth microdilutions were added to the seeded HepG2-containing 384-well plates and incubated at 37 °C for another 48 h. After 48 h incubation, a 1:4 dilution of Alamar Blue cell viability reagent (Invitrogen) in DMEM-FBS was added to each well to a final concentration of 1:20. Plates were incubated at 37 °C for 2 h and fluorescence was read using a microplate fluorometer (Tecan Infinite M1000Pro, 530 nm excitation and 590 nm emission). All assays were done in triplicate and are representative of biological duplicates.

To quantitatively assess NPD827 efficacy in an in vitro co-culture experiment, 293T cells stably expressing firefly luciferase were plated in 384-well format (2000 cells in 20 μL/well, black clear-bottom plates) and allowed to adhere overnight. Wells were then infected with log-phase GFP-marked CaCi-2 or CaCi-17 (2.5 × 10^4^ cells/mL) in an equal volume of medium supplemented with fluconazole (4 μg/mL). After 48 h, medium was replaced with PBS and relative fluorescence per well measured using a microplate fluorometer (Tecan Infinite M1000Pro, 480 nm excitation and 540 nm emission). Steady-Glo luciferase assay reagent (10 μL/well, Promega Cat# E2510) was then added, plates incubated at room temperature for 10 min and relative luminescence per well measured (Tecan Infinite M1000Pro).

To examine the effects of lovastatin on NPD827 toxicity, the experiment was performed as described in methods for 293T cells except assay plates were inoculated with 2000 cells/well. A titration of lovastatin (Sigma) was added as well as NPD827. Plates were incubated for 48 h and a 1:4 dilution of Alamar Blue cell viability reagent (Invitrogen) in PBS was added to each well to a final concentration of 1:20. Plates were incubated at 37 °C for 3 h and fluorescence was read using a microplate fluorometer (Tecan Infinite M1000Pro, 530 nm excitation and 590 nm emission). All assays were done in triplicate and are representative of biological duplicates. Data was analyzed by subtracting background fluorescence plotted in Prism v9.3.0 (GraphPad) showing the mean fluorescence ± SD. Synergy between NPD827 and lovastatin was evaluated using SynergyFinder (https://synergyfinder.fimm.fi) which found a ZIP score of −3.278, indicating no synergy.

### *C. elegans* infection assay

*C. elegans* strains N2 (wild-type) and AU37 (glp-4(bn2);sek-1(km4)) were obtained from the Caenorhabditis Genetics Center (CGC) and were maintained on superfood nematode growth media (NGM) with *E. coli* (OP50) at 15 °C, as previously described^41^. After 5 days, worms were transferred to room temperature (20–25 °C) for one day to synchronize growth. Cultures of C. albicans clinical isolates CaCi-2 and CaCi-17 were incubated overnight at 30 °C in YPD with shaking. Overnight cultures of *C. albicans* were plated onto brain heart infusion (BHI) agar plates containing 50 µg/mL kanamycin by transferring 10 µL of each culture spread over the surface using a plate spreader, and allowed to grow at 30 °C for 16 h. A liquid media-based *C. elegans* infection assay protocol was used, based on previous studies^83–85^. Briefly, 48-well plates were prepared with each well containing either: fluconazole (16 µg/mL for CaCi-2 or 256 µg/mL for CaCi-17), NPD827 (40 µg/mL), fluconazole and NPD827 (16 or 256 µg/mL fluconazole with 40 µg/mL NPD827), or no drug treatment. *C. elegans* were washed twice from NGM plates using 6 mL of M9 buffer with swirling to dislodge worms from agar and transferred to a 15 mL conical tube. Conical tubes with worms were centrifuged at 1500×*g* for 30 s, buffer was removed, and worms were washed twice with additional M9 buffer. The worms were counted under a stereomicroscope at ×10 magnification, and the concentration of worms per microliter of solution was calculated, as previously described^41^. The worm solution was adjusted to 1 worm/µL, and 100 worms were added to BHI + kanamycin plates, and allowed to feed for 3 h. The worms were then rinsed off plates with 500 µL of M9 buffer, transferred to a 15 mL conical tube, 10 mL of additional M9 buffer was added, and the worms were centrifuged at 1500×*g* for 30 s and washed twice. The worm pellet was resuspended in 300 µL of M9, and worms were allowed to crawl on unseeded BHI plates for 30 min. Worms were rinsed from plate and transferred to a new conical tube with 1 mL of M9 buffer, the number of worms in the sample was determined once again and adjusted to 1 worm/µL. 20 worms (20 µL of sample) was then added in to each well of the prepared 48-well plates. Plates were incubated at room temperature overnight, and worm survival was scored at 16, 24, and 48 h under a stereomicroscope at ×10 magnification.

### Plasmid construction

Plasmid construction was performed using standard recombinant DNA techniques. Plasmids were sequenced to verify the absence of spurious mutations. Primers are listed in Supplementary Table 3.

#### pLC1446

From *erg3Δ/erg3Δ* (CaLC7094) genomic DNA, ~300 bp of homology downstream of *VPS4* was amplified with oLC8797/8798 and cloned into pLC49 (pJK863) at SacI and SacII. Homology from upstream and within *VPS4* was amplified from *erg3Δ/erg3Δ* (CaLC7094) genomic DNA using oLC8795/8796 and cloned into pLC49 containing the downstream homology at ApaI and KpnI. Presence of the inserts was confirmed with oLC274/oLC8800 and oLC8799/oLC275, respectively. The *VPS4*^WT^ allele replacement cassette was liberated by digestion with BssHII.

#### pLC1447

From *erg3Δ/erg3Δ* (CaLC7094) genomic DNA, ~600 bp of homology downstream of *VPS20* was amplified with oLC8805/8806 and cloned into pLC49 (pJK863) at SacI and SacII. Homology from upstream and within *VPS20* was amplified from *erg3Δ/erg3Δ* (CaLC7094) genomic DNA using oLC8803/oLC8804 and cloned into pLC49 containing the downstream homology at ApaI and KpnI. Presence of the inserts was confirmed with oLC274/oLC8800 and oLC8799/oLC275, respectively. The *VPS20*^WT^ allele replacement cassette was liberated by digestion with BssHII.

#### pLC1442

From *erg3Δ/erg3Δ*-RM1 (CaLC7095) genomic DNA, ~300 bp of homology downstream of *VPS4* was amplified with oLC8797/8798 and cloned into pLC49 (pJK863) at SacI and SacII. Homology from upstream and within *VPS4* was amplified from RM1 (CaLC7095) genomic DNA using oLC8795/8796 and cloned into pLC49 containing the downstream homology at ApaI and KpnI. Presence of the inserts was confirmed with oLC274/oLC8800 and oLC8799/oLC275, respectively. The *VPS4*^D234Y^ allele replacement cassette was liberated by digestion with BssHII.

#### pLC1444

From *erg3Δ/erg3Δ*-RM4 (CaLC7098) genomic DNA, ~300 bp of homology downstream of *VPS4* was amplified with oLC8797/8798 and cloned into pLC49 (pJK863) at SacI and SacII. Homology from upstream and within *VPS4* was amplified from RM4 (CaLC7098) genomic DNA using oLC8795/8796 and cloned into pLC49 containing the downstream homology at ApaI and KpnI. Presence of the inserts was confirmed with oLC274/oLC8800 and oLC8799/oLC275, respectively. The *VPS4*^G178E^ allele replacement cassette was liberated by digestion with BssHII.

#### pLC1445

From *erg3Δ/erg3Δ*-RM6 (CaLC7100) genomic DNA, ~600 bp of homology downstream of *VPS20* was amplified with oLC8805/8806 and cloned into pLC49 (pJK863) at SacI and SacII. Homology from upstream and within *VPS20* was amplified from RM6 (CaLC7100) genomic DNA using oLC8803/oLC8804 and cloned into pLC49 containing the downstream homology at ApaI and KpnI. Presence of the inserts was confirmed with oLC274/oLC8800 and oLC8799/oLC275, respectively. The *VPS20*^E194*^ allele replacement cassette was liberated by digestion with BssHII.

### Strain construction

Strains were constructed according to standard protocols. To select for nourseothricin (NAT)-resistant transformants, nourseothricin (Jena Bioscience) was solubilized in water and supplemented into YPD plates at a final concentration of 150 µg/mL.

#### CaLC7113

The plasmid pLC1446 was digested with BssHII to liberate the *VPS4*^WT^ allele replacement cassette and was transformed into *erg3Δ/erg3Δ*-RM1 (CaLC7095). For NAT resistant transformants, proper integration was verified by PCR using oLC8799/oLC275 and oLC274/oLC8802. The *SAP2* promoter was induced to drive expression of FLP recombinase to excise the NAT marker cassette. Replacement of the mutant allele of *VPS4* with the wild type was confirmed with oLC8799 by sequencing a PCR product amplified by oLC8787/oLC8788.

#### CaLC7114

The plasmid pLC1446 was digested with BssHII to liberate the *VPS4*^WT^ allele replacement cassette and was transformed into *erg3Δ/erg3Δ*-RM4 (CaLC7098). For NAT resistant transformants, proper integration was verified by PCR using oLC8799/oLC275 and oLC274/oLC8802. The *SAP2* promoter was induced to drive expression of FLP recombinase to excise the NAT marker cassette. Replacement of the mutant allele of *VPS4* with the wild type was confirmed with oLC8799 by sequencing a PCR product amplified by oLC8787/oLC8788.

#### CaLC7115

The plasmid pLC1447 was digested with BssHII to liberate the *VPS20*^WT^ allele replacement cassette and was transformed into *erg3Δ/erg3Δ*-RM6 (CaLC7100). For NAT resistant transformants, proper integration was verified by PCR using oLC8807/oLC275 and oLC274/oLC8808. The *SAP2* promoter was induced to drive expression of FLP recombinase to excise the NAT marker cassette. Replacement of the mutant allele of *VPS20* with the wild type was confirmed with oLC8789 by sequencing a PCR product amplified by oLC8807/oLC275.

#### CaLC7110

The plasmid pLC1442 was digested with BssHII to liberate the *VPS4*^D234Y^ allele replacement cassette and was transformed into *erg3Δ/erg3Δ* (CaLC660). For NAT resistant transformants, proper integration was verified by PCR using oLC8799/oLC275 and oLC274/oLC8802. The *SAP2* promoter was induced to drive expression of FLP recombinase to excise the NAT marker cassette.

#### CaLC7111

The plasmid pLC1444 was digested with BssHII to liberate the *VPS4*^G178E^ allele replacement cassette and was transformed into *erg3Δ/erg3Δ* (CaLC660). For NAT resistant transformants, proper integration was verified by PCR using oLC8799/oLC275 and oLC274/oLC8802. The *SAP2* promoter was induced to drive expression of FLP recombinase to excise the NAT marker cassette.

#### CaLC7112

The plasmid pLC1445 was digested with BssHII to liberate the *VPS20*^E194*^ allele replacement cassette and was transformed into *erg3Δ/erg3Δ* (CaLC660). For NAT resistant transformants, proper integration was verified by PCR using oLC8807/oLC275 and oLC274/oLC8808. The *SAP2* promoter was induced to drive expression of FLP recombinase to excise the NAT marker cassette.

#### CaLC7486

Transient CRISPR was used to generate *vps4∆/vps4∆* in SN95 (CaLC239). Cas9 was amplified from pLC963 using oLC6924/oLC6925. The guide was generated using fusion PCR. Amplicons were generated from pLC963 with oLC5978/oLC9763 and oLC5980/oLC9762 and fused together using oLC5981/oLC5879. Repair was amplified using oLC9764/oLC9765 from XmaI-digested pLC49. All three pieces (Cas9, guide, repair) were transformed into SN95 (CaLC239). For NAT resistant transformants, proper integration and absence of wild type *VPS4* was verified by PCR using oLC8801/oLC275 and oLC8800/oLC8801, respectively.

#### CaLC7487

Transient CRISPR was used to generate *vps4∆/vps4∆* in an *erg3∆/erg3∆* (CaLC660) background. Cas9 was amplified from pLC963 using oLC6924/oLC6925. The guide was generated using fusion PCR. Amplicons were generated from pLC963 with oLC5978/oLC9763 and oLC5980/oLC9762 and fused together using oLC5981/oLC5879. Repair was amplified using oLC9764/oLC9765 from XmaI-digested pLC49. All three pieces (Cas9, guide, repair) were transformed into *erg3∆/erg3∆* (CaLC660). For NAT resistant transformants, proper integration and absence of wild type *VPS4* was verified by PCR using oLC8801/oLC275 and oLC8800/oLC8801, respectively.

#### CaLC7488

Transient CRISPR was used to generate *vps20∆/vps20∆* in SN95 (CaLC239). Cas9 was amplified from pLC963 using oLC6924/oLC6925. The guide was generated using fusion PCR. Amplicons were generated from pLC963 with oLC5978/oLC9768 and oLC5980/oLC9767 and fused together using oLC5981/oLC5879. Repair was amplified using oLC9769/oLC9770 from XmaI-digested pLC49. All three pieces (Cas9, guide, repair) were transformed into SN95 (CaLC239). For NAT resistant transformants, proper integration and absence of wild type *VPS20* was verified by PCR using oLC8809/oLC275 and oLC8808/oLC8809, respectively.

#### CaLC7489

Transient CRISPR was used to generate *vps20∆/vps20∆* in an *erg3∆/erg3∆* (CaLC660) background. Cas9 was amplified from pLC963 using oLC6924/oLC6925. The guide was generated using fusion PCR. Amplicons were generated from pLC963 with oLC5978/oLC9768 and oLC5980/oLC9767 and fused together using oLC5981/oLC5879. Repair was amplified using oLC9769/oLC9770 from XmaI-digested pLC49. All three pieces (Cas9, guide, repair) were transformed into *erg3∆/erg3∆* (CaLC660). For NAT resistant transformants, proper integration and absence of wild type *VPS20* was verified by PCR using oLC8809/oLC275 and oLC8808/oLC8809, respectively.

### Reporting summary

Further information on research design is available in the Nature Research Reporting Summary linked to this article.

## Supplementary information

Supplementary Information

Reporting Summary

## Data Availability

All data generated or analyzed during this study are included in this published article (and its supplementary information files), or are available from the corresponding author upon reasonable request. Source data are provided with this paper.

## Source data

Source Data
