# Supplementary material for: Targeting fungal membrane homeostasis with imidazopyrazoindoles impairs azole resistance and biofilm formation
Source: Nat Commun. 2022 Jun 25;13:3634. doi: 10.1038/s41467-022-31308-1 (PMC9233667; doi:10.1038/s41467-022-31308-1)
Supplement: Supplementary file 1 — Supplementary Information [file 41467_2022_31308_MOESM1_ESM.pdf]

Supplementary Information

Targeting fungal membrane homeostasis with imidazopyrazoindoles impairs azole resistance and biofilm formation

Nicole M. Revie, Kali R. Iyer, Michelle E. Maxson, Jiabao Zhang, Su Yan, Caroline M. Fernandes, Kirsten J. Meyer, Xuefei Chen, Iwona Skulska, Meea Fogal, Hiram Sanchez, Saif Hossain, Sheena Li, Yoko Yashiroda, Hiroyuki Hirano, Minoru Yoshida, Hiroyuki Osada, Charles Boone, Rebecca S. Shapiro, David R. Andes, Gerard D. Wright, Justin R. Nodwell, Maurizio Del Poeta, Martin D. Burke, Luke Whitesell, Nicole Robbins, and Leah E. Cowen

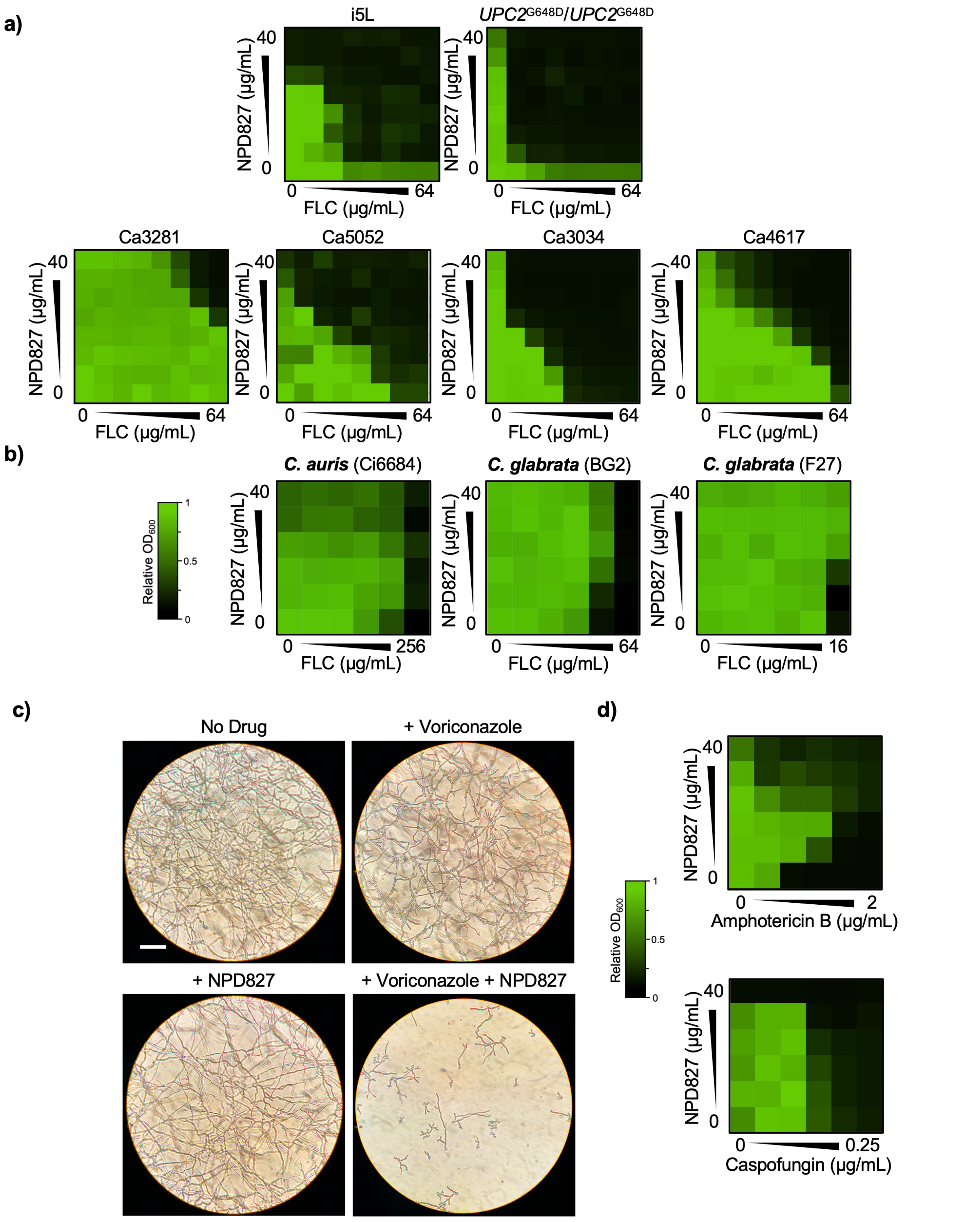

Supplementary Fig. 1. NPD827 potentiates ergosterol biosynthesis inhibitors against select fungal pathogens. a. Checkerboard assays were performed against a panel of *C. albicans* isolates.

These assays were performed in YPD medium at 30°C and growth was measured using absorbance at 600 nm after 24 hours. Measurements were normalized to no drug controls and data was quantitatively displayed with colour using Treeview (see colour bar). Experiments were performed in biological duplicate. **b.** Checkerboard assays were performed against a clinical isolate of *C. auris* (Ci6684) and two isolates of *C. glabrata* (BG2 and F27). These assays were performed in YPD medium at 30°C and growth was measured using absorbance at 600 nm after 24 hours. Measurements were normalized to no drug controls and data was quantitatively displayed with colour using Treeview (see colour bar). Experiments were performed in biological duplicate. **c.** Checkerboard assays were performed against *A. fumigatus* (Af239) as shown in Fig. 1c. Images were taken using a light microscope after 48 hours of growth at 37°C. Scale bar is 100 µm. Voriconazole, 0.125 µg/mL, NPD827 = 10 µg/mL. Experiments were performed in biological duplicate. **d.** Checkerboard assays were performed against wild type *C. albicans* (SN95) using an ergosterol extracting antifungal (AmpB), and a cell wall-targeting agent (Caspofungin). These assays were performed in YPD medium at 30°C and growth was measured using absorbance at 600 nm after 24 hours. Measurements were normalized to no drug controls and data was quantitatively displayed with colour using Treeview (see colour bar). Experiments were performed in biological duplicate. Source data are provided as a Source Data file.

a)

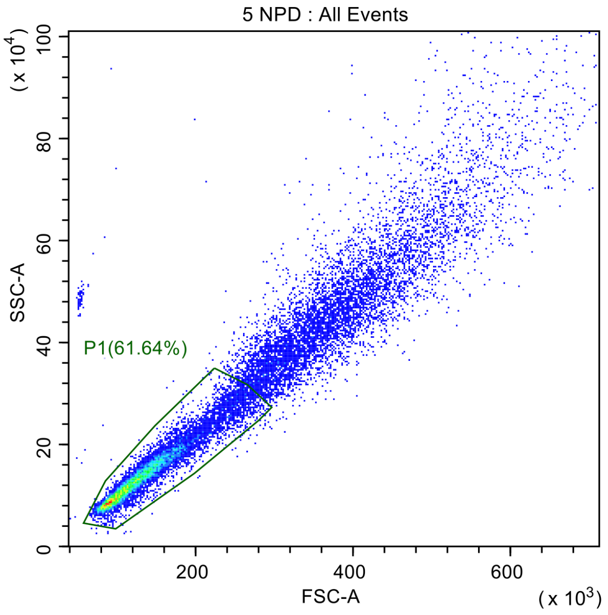

b)

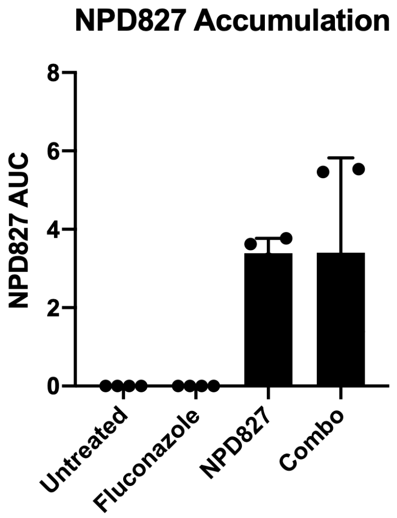

Supplementary Fig. 2. NPD827 alters membrane dynamics and perturbs multi-drug efflux. a. Pseudo-coloured density plots displaying the side-scatter and forward-scatter data for each even recorded in an untreated sample of *C. albicans* cells when run on the Cytoflex flow cytometer and analyzed with the CytExpert Software (version 2.4). Gates displayed in boundary are the

gates that were applied to all samples for data shown in Fig. 2b. **b.** Relative intracellular concentrations of NPD827 were measured after treatment for 1 hour in the presence and absence of fluconazole. Data are presented as mean ± SD of technical triplicates. Source data are provided as a Source Data file.

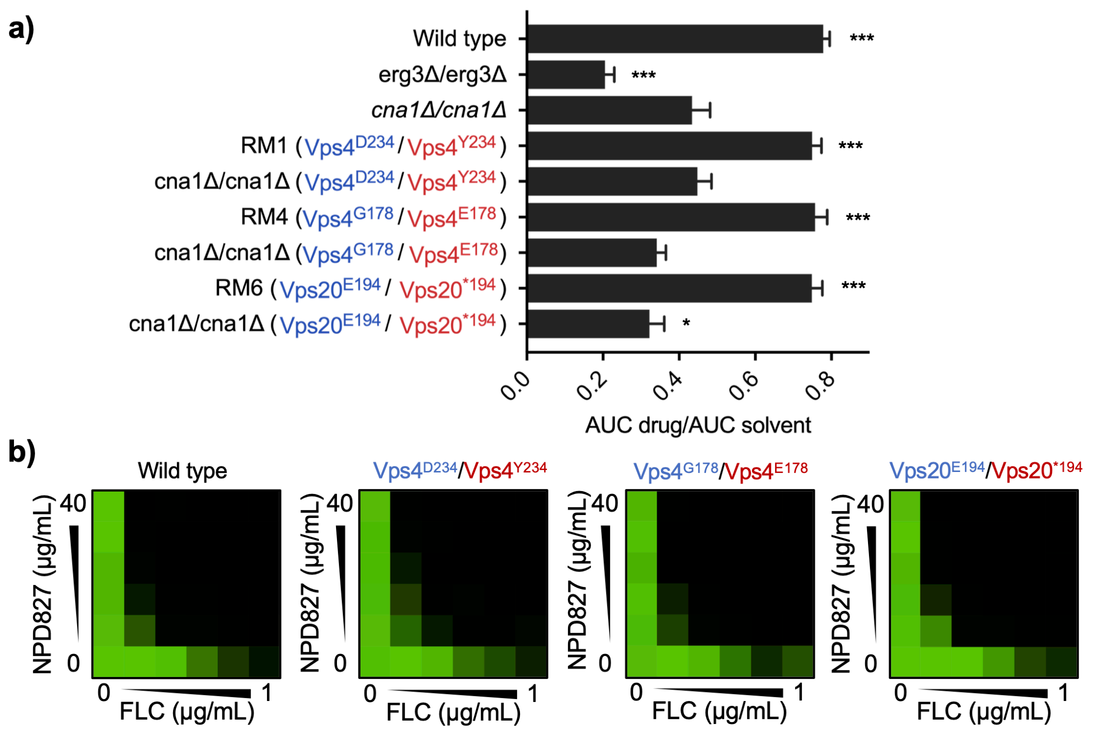

Supplementary Fig. 3. NPD827 resistant mutations, identified in an *erg3Δ/Δ* background, do not confer resistance in hypersensitive *cna1Δ/Δ* or a wild-type strain of *C. albicans*. a. Allele swap strains in a *cna1Δ/Δ* background were assessed by growth curve analysis in the absence or presence of NPD827 (20 µg/mL) for 48 hours in YPD at 30°C, and growth was measured every 15 min by absorbance at 600 nm. The area under the resulting growth curves (AUC) were calculated and the ratio of $AUC_{drug}/AUC_{solvent}$ was normalized relative to a wild-type control. Data are expressed as mean ± SD of n = 3 biologically independent replicates. Significance determined by one-way ANOVA with Bonferroni's multiple comparisons test, where all conditions were compared to the parent *(cna1Δ/Δ)*, *** <0.0005 p-value. P-value, compared to *cna1Δ/cna1Δ* parent: <0.0001 (Wildtype, *erg3Δ/erg3Δ,* RM1 [$Vps4^{D234/Y234}$], RM4 [$Vps4^{G178/E178}$], RM6 [$Vps20^{E194/*194}$]). **b.** Checkerboard assays were performed with NPD827 and fluconazole against a wild-type strain with heterozygous mutations in *VPS4* and *VPS20*. In the wild-type (SN95)

control, the following Vps4 amino acids are present at both alleles: D234, G178, and E194. These assays were performed in YPD medium at 30°C and growth was measured using absorbance at 600 nm after 24 hours. Measurements were normalized to no drug controls and data was quantitatively displayed with colour using Treeview (see colour bar). Experiments were performed in biological duplicate. Parental allele is displayed in blue, resistant allele is displayed in red. Source data are provided as a Source Data file.

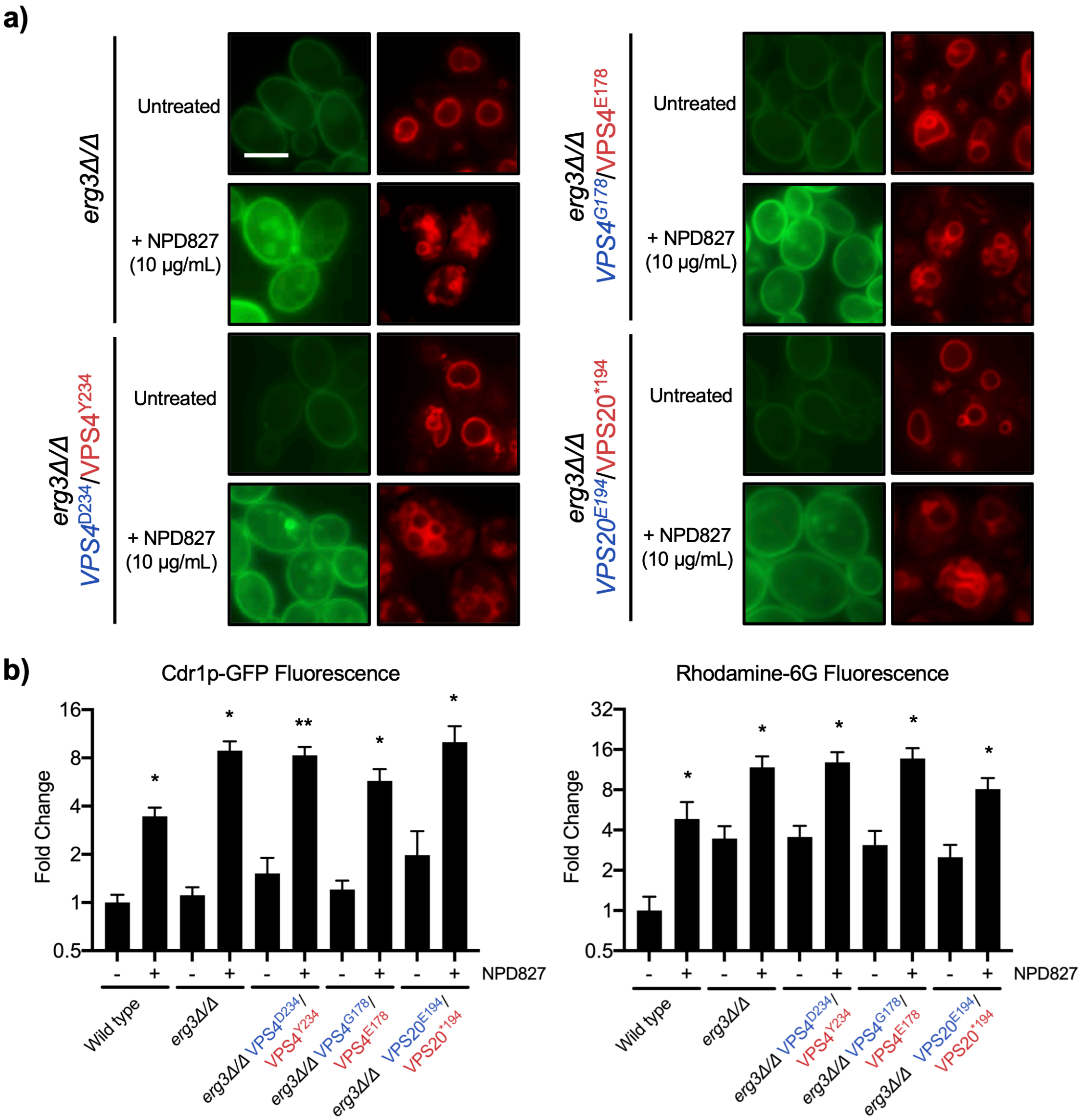

Supplementary Fig 4. NPD827 activity impairs Cdr1 function in an *erg3Δ/Δ* mutant and corresponding resistant mutants. a. Cdr1-GFP localization and FM4-64 staining upon NPD827 treatment (10 µg/mL). Differential interference contrast (DIC) and fluorescence microscopy imaging with GFP and RFP filters. Assays were performed in biological triplicate. Scale bar represents 5 µm. Parental allele is displayed in blue, resistant allele is displayed in red. **b.** Quantification of Cdr1p-GFP and Rhodamine-6G fluorescence upon NPD827 treatment. For Cdr1p-GFP assays, *C. albicans* cells were grown for 30 minutes with NPD827 (10 µg/mL). For

Rhodamine-6G assays, *C. albicans* cells were grown for 30 minutes with NPD827 (10 µg/mL) before staining with rhodamine-6G (1 µg/mL). Assays were performed in biological duplicate. Bars represent median fluorescence, normalized to untreated cells, as quantified by flow cytometry for at least 20,000 events. Error bars represent standard deviation across biological triplicates. Significance was determined by performing a two-tailed unpaired t test with Welch's correction, where all conditions were compared to their corresponding untreated controls*,* p-value: * <0.05 p-value, * <0.05 p-value, ** <0.005 p-value, *** <0.0005 p-value. Parental allele is displayed in blue, resistant allele is displayed in red. P-value, compared to corresponding untreated controls: Cdr1p-GFP, 0.0044 (Wildtype); 0.0045 (*erg3Δ/erg3*Δ); 0.0008 (*erg3Δ/erg3*Δ Vps4$^{D234/Y234}$); 0.0118 (*erg3Δ/erg3*Δ Vps4$^{G178/E178}$); 0.0205 (*erg3Δ/erg3*Δ Vps20$^{E194/*194}$); R6G, 0.0373 (Wildtype); 0.0007 (*erg3Δ/erg3*Δ); 0.0003 (*erg3Δ/erg3*Δ Vps4$^{D234/Y234}$); 0.0005 (*erg3Δ/erg3*Δ Vps4$^{G178/E178}$); 0.0010 (*erg3Δ/erg3*Δ Vps20$^{E194/*194}$). Source data are provided as a Source Data file.

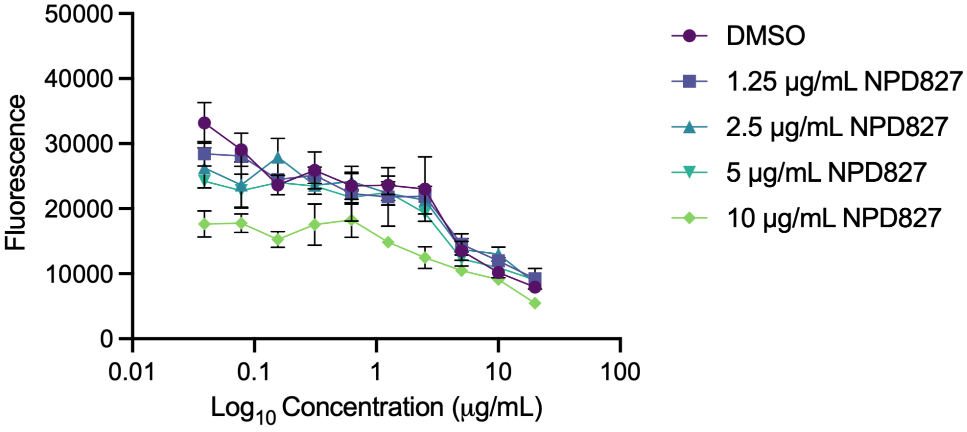

Supplementary Fig. 5. The addition of NPD827 does not affect lovastatin toxicity in mammalian cells. Chemical susceptibility assays were performed with 293T cells that expressed firefly luciferase. Cells were seeded overnight with 2,000 cells/well in DMEM medium before the indicated background concentrations of NPD827 and a 2-fold titration of lovastatin was added to each well. Plates were incubated for 48 hours at 37 °C before cell viability was measured using the fluorescent metabolic dye Alamar Blue. Bars represent median fluorescence, +/- SD of triplicate measurements. Assays were performed in biological duplicate with representative results shown. Synergy between NPD827 and lovastatin was evaluated using SynergyFinder (https://synergyfinder.fimm.fi), which found a ZIP score of -3.278, indicating no synergy. Source data are provided as a Source Data file.

Supplementary Table 1. Strains used in this manuscript.

Name	Alias	Genotype	Source
CaLC79	Early C.I. (CaCi-2)	Prototrophic (Clinical Isolate)	1
CaLC91	Late C.I. (CaCi-17)	Prototrophic (Clinical Isolate)	1
CaLC155	*C. albicans* SC5314	Prototrophic	2
CaLC176	*C. albicans* Clinical isolate Ca3281	Prototrophic	3,4
CaLC177	*C. albicans* Clinical isolate Ca5052	Prototrophic	3,4
CaLC180	*C. albicans* Clinical isolate Ca3034	Prototrophic	3,4
CaLC182	*C. albicans* Clinical isolate Ca4617	Prototrophic	3,4
CaLC188	*cnb1Δ/cnb1Δ*	*ura3::imm434/ura3::imm434 his1::hisG::HIS1/his1::hisG arg4::hisG/arg4::hisG cnb1::UAU1/cnb1::ARG4*	5
CaLC190	*cnb1Δ/cnb1Δ* + *CNB1*	*ura3::imm434/ura3::imm434 his1::hisG::CNB1-HIS1/his1::hisG arg4::hisG/arg4::hisG cnb1::UAU1/cnb1::ARG4*	5
CaLC239	*C. albicans* SN95 Wild type	*arg4/arg4 his1/his1 URA3/ura3::imm434 IRO1/iro1::imm434*	6
CaLC563	CAI4 *efg1Δ/efg1Δ*	*ura3::imm434/ura3::imm434 efg1::hisG/efg1::hisG-URA3-hisG*	7
CaLC867	CaCi-2 *ENO1*-GFP	As CaLC79, ENO1-GFP-NAT/ENO1	8
CaLC868	CaCi-17 *ENO1*-GFP	As CaLC91, ENO1-GFP-NAT/ENO1	8
CaLC877	D11-3.3 + i5L	Prototrophic	9
CaLC908	*CNA1/cna1Δ*	As SN95, *CNA1/cna1::FRT*	10
CaLC909	*cna1Δ/cna1Δ*	As SN95, *cna1::FRT/cna1::FRT*	10
CgLC1002	*C. glabrata* BG2	Clinical Isolate	11
CaLC1446	*mid1Δ/mid1Δ*	As SC5314, *mid1::FRT/mid1::FRT*	12
CaLC1448	*cch1Δ/cch1Δ*	As SC5314, *cch1::FRT/cch1::FRT*	12
CaLC1449	*mid1Δ/mid1Δ cch1Δ/cch1Δ*	As SC5314, *mid1::FRT/mid1::FRT cch1::FRT/cch1::FRT*	12
CaLC1776	SC5314, $UPC2^{G648D}/UPC2^{G648D}$	As CaLC155, $UPC2^{G648D}$*::FRT*/$UPC2^{G648D}$*::FRT*	13
CaLC2751	Ca-*cna1/CNA1*	*cnaD::hisG/cnaD::hisG LEU2::CNA::URA3*	14
CaLC2752	Ca-*cna1/CNA1tr*	*cnaD::hisG/cnaD::hisG LEU2::CNAtr::URA3*	14
CnLC3142	*Cryptococcus neoformans* Reference Serotype A	Clinical Isolate	15

CaLC3365	*C. albicans* CaSS1	*arg4/arg4 his1/his1 URA3/ura3::imm434 IRO1/iro1 ::imm434*	[16]
AfLC3669	*Aspergillus fumigatus* reference strain Af293	Clinical Isolate	[17]
CauLC5083	*Candida auris* Ci6684	Clinical Isolate Clade I	[18]
CaLC6106	*C. albicans* GRACE parent strain	*ura3::imm434/ura3::imm434 his3::hisG/his3::hisG leu2::tetRGAL4AD-URA/LEU2*	[16]
CaLC7095	NPD827 Resistant Isolate #1 (*erg3*Δ*/ erg3*Δ-RM1)	As CaLC660, NPD827 resistant	This study
CaLC7096	NPD827 Resistant Isolate #2 (*erg3*Δ *erg3/*Δ-RM2)	As CaLC660, NPD827 resistant	This study
CaLC7097	NPD827 Resistant Isolate #3 (*erg3*Δ*/ erg3*Δ-RM3)	As CaLC660, NPD827 resistant	This study
CaLC7098	NPD827 Resistant Isolate #4 (*erg3*Δ*/ erg3*Δ-RM4)	As CaLC660, NPD827 resistant	This study
CaLC7099	NPD827 Resistant Isolate #5 (*erg3*Δ*/ erg3*Δ-RM5)	As CaLC660, NPD827 resistant	This study
CaLC7100	NPD827 Resistant Isolate #6 (*erg3*Δ*/ erg3*Δ-RM6)	As CaLC660, NPD827 resistant	This study
CaLC7110	*erg3*Δ*/erg3*Δ + *VPS4/VPS4*^{D234Y}	As CaLC660, *VPS4/VPS4*^{D234Y}::FRT	This study
CaLC7111	*erg3*Δ*/erg3*Δ + *VPS4/VPS4*^{G178E}	As CaLC660, *VPS4/VPS4*^{G178E}::FRT	This study
CaLC7112	*erg3*Δ*/erg3*Δ + *VPS20/VPS20*^{E194*}	As CaLC660, *VPS20/VPS20*^{E194*}::FRT	This study
CaLC7113	*erg3*Δ*/erg3*Δ-RM1 + *VPS4/VPS4*^{WT}	As CaLC7095, *VPS4/VPS4*::FRT	This study
CaLC7114	*erg3*Δ*/erg3*Δ-RM4 + *VPS4/VPS4*^{WT}	As CaLC7098, *VPS4/VPS4*::FRT	This study
CaLC7115	*erg3*Δ*/erg3*Δ-RM6 + *VPS20/VPS20*^{WT}	As CaLC7100, *VPS20/VPS20*::FRT	This study
CaLC7116	Wild type *CDR1*-GFP	As CaLC239, *CDR1/CDR1*-GFP-NAT	This study
CaLC7464	Wild type GFP-*RAS1*	As CaLC239, tetO-GFP-*RAS1/RAS1*	This study
CaLC7486	*vps4*Δ*/vps4*Δ	As CaLC239, *vps4*Δ*/vps4*Δ	This study
CaLC7487	*erg3*Δ*/erg3*Δ *vps4*Δ*/vps4*Δ	As CaLC660, *vps4*Δ*/vps4*Δ	This study
CaLC7488	*vps20*Δ*/vps20*Δ	As CaLC239, *vps20*Δ*/vps20*Δ	This study
CaLC7489	*erg3*Δ*/erg3*Δ *vps20*Δ*/vps20*Δ	As CaLC660, *vps20*Δ*/vps20*Δ	This study
GRACE strain	*tetO-ERG10/erg10*Δ	As CaLC6106, *SAT1::tetO-ERG10/erg10::HIS3*	[16]
GRACE strain	*tetO-ERG13/erg13*Δ	As CaLC6106, *SAT1::tetO-ERG13/erg13::HIS3*	[16]
GRACE strain	*tetO-HMG1/hmg1*Δ	As CaLC6106, *SAT1::tetO-HMG1/hmg1::HIS3*	[16]
GRACE strain	*tetO-ERG12/erg12*Δ	As CaLC6106, *SAT1::tetO-ERG12/erg12::HIS3*	[16]
GRACE strain	*tetO-ERG8/erg8*Δ	As CaLC6106, *SAT1::tetO-ERG8/erg8::HIS3*	[16]
GRACE strain	*tetO-ERG19/erg19*Δ	As CaLC6106, *SAT1::tetO-ERG19/erg19::HIS3*	[16]

GRACE strain	*tetO-IDI1/idi1Δ*	As CaLC6106, *SAT1::tetO-IDI1/idi1::HIS3*	[16]
GRACE strain	*tetO-ERG20/erg20Δ*	As CaLC6106, *SAT1::tetO-ERG20/erg20::HIS3*	[16]
GRACE strain	*tetO-ERG1/erg1Δ*	As CaLC6106, *SAT1::tetO-ERG1/erg1::HIS3*	[16]
GRACE strain	*tetO-ERG7/erg7Δ*	As CaLC6106, *SAT1::tetO-ERG7/erg7::HIS3*	[16]
GRACE strain	*tetO-ERG11/erg11Δ*	As CaLC6106, *SAT1::tetO-ERG11/erg11::HIS3*	[16]
GRACE strain	*tetO-ERG24/erg24Δ*	As CaLC6106, *SAT1::tetO-ERG24/erg24::HIS3*	[16]
GRACE strain	*tetO-ERG6/erg6Δ*	As CaLC6106, *SAT1::tetO-ERG6/erg6::HIS3*	[16]
GRACE strain	*tetO-ERG27/erg27Δ*	As CaLC6106, *SAT1::tetO-ERG27/erg27::HIS3*	[16]
GRACE strain	*tetO-ERG2/erg2Δ*	As CaLC6106, *SAT1::tetO-ERG2/erg2::HIS3*	[16]
GRACE strain	*tetO-ERG3/erg3Δ*	As CaLC6106, *SAT1::tetO-ERG3/erg3::HIS3*	[16]
GRACE strain	*tetO-ERG5/erg5Δ*	As CaLC6106, *SAT1::tetO-ERG5/erg5::HIS3*	[16]
GRACE strain	*tetO-ERG4/erg4Δ*	As CaLC6106, *SAT1::tetO-ERG4/erg4::HIS3*	[16]
GRACE strain	*tetO-LCB1/lcb1Δ*	As CaLC6106, *SAT1::tetO-LCB1/lcb1::HIS3*	[16]
GRACE strain	*tetO-LCB2/lcb2Δ*	As CaLC6106, *SAT1::tetO-LCB2/lcb2::HIS3*	[16]
GRACE strain	*tetO-KSR1/ksr1Δ*	As CaLC6106, *SAT1::tetO-KSR1/ksr1::HIS3*	[16]
GRACE strain	*tetO-LAG1/lag1Δ*	As CaLC6106, *SAT1::tetO-LAG1/lag1::HIS3*	[16]
GRACE strain	*tetO-LIP1/lip1Δ*	As CaLC6106, *SAT1::tetO-LIP1/lip1::HIS3*	[16]
GRACE strain	*tetO-SLD1/sld1Δ*	As CaLC6106, *SAT1::tetO-SLD1/sld1::HIS3*	[16]
GRACE strain	*tetO-MTS1/mts1Δ*	As CaLC6106, *SAT1::tetO-MTS1/mts1::HIS3*	[16]
GRACE strain	*tetO-SUR2/sur2Δ*	As CaLC6106, *SAT1::tetO-SUR2/sur2::HIS3*	[16]
GRACE strain	*tetO-SCS7/scs7Δ*	As CaLC6106, *SAT1::tetO-SCS7/scs7::HIS3*	[16]
GRACE strain	*tetO-AUR1/aur1Δ*	As CaLC6106, *SAT1::tetO-AUR1/aur1::HIS3*	[16]
GRACE strain	*tetO-CSG2/csg2Δ*	As CaLC6106, *SAT1::tetO-CSG2/csg2::HIS3*	[16]
GRACE strain	*tetO-IPT1/ipt1Δ*	As CaLC6106, *SAT1::tetO-IPT1/ipt1::HIS3*	[16]

Supplementary Table 2. Plasmids used in this manuscript.

Name	Description	Source
pLC49	p863, FLP-CaNAT, ampR	[19]
pLC963	pV1393-1 (CaCas9/sgRNA entry expression vector, contains NatR gene, targeting NEUT5L)	[20]
pLC1442	p863, Ca*VPS4*^{D234Y}, FLP-CaNAT, ampR	This study
pLC1444	p863, Ca*VPS4*^{G178E}, FLP-CaNAT, ampR	This study
pLC1445	p863, Ca*VPS20*^{E194*}, FLP-CaNAT, ampR	This study
pLC1446	p863, Ca*VPS4*, FLP-CaNAT, ampR	This study
pLC1447	p863, Ca*VPS20*, FLP-CaNAT, ampR	This study

pLC1470	pLC605-GFP	This study

Supplementary Table 3. Oligonucleotides used in this manuscript.

Name	Description	Sequence (5'-3')
oLC274	pJK863down-F	CTGTCAAGGAGGGTATTCTGG
oLC275	pJK863up-R	AAAGTCAAAGTTCCAAGGGG
oLC4345	Ca*HAC1*+1113-R	TGAACTTCAACATCATCTCC
oLC4347	Ca*HAC1*+885-F	CCCATCACCACTTTCATTCC
oLC5978	pLC963-SNR52-F	GACTGTCAAGGAGGGTATTC
oLC5979	pLC963-SNR52-N-F	CCGCAACTGATTAGACTTAG
oLC5980	pLC963-sgRNA-R	GAATACCACTTGTTTACCGG
oLC5981	pLC963-sgRNA-N-R	GGTGGCGGCAAAACTAATTC
oLC6924	CaCas9/for	ATCTCATTAGATTTGGAACTTGTGGGTT
oLC6925	CaCas9/rev	TTCGAGCGTCCCAAAACCTTCT
oLC8787	Ca*VPS4*+311-F	TCTAATGGTGATGGCAATGG
oLC8788	Ca*VPS4*+1191-R	TTCATCAGTGGCAAGATCCATCC
oLC8789	Ca*VPS20*+497-F	ACATGGCAAAATCAAACAGG
oLC8795	Ca*VPS4*-97_KpnI-F	TTGCGGTACCCAACTAAAATTAATAACGCCACCTCC
oLC8796	Ca*VPS4*+1320_ApaI-R	TTGCGGGCCCTTAATTACCTTCTTGACCGAAATCTTCTG
oLC8797	Ca*VPS4*+1320_SacII-F	TTGCCCGCGGAAGAGGGGCCAATGAATAAAATATATTC
oLC8798	Ca*VPS4*+1699_SacI-R	TTGCGAGCTCTCTAGAAGAAAGAATGAGGGGAATGACG
oLC8799	Ca*VPS4*+421-F	GGTCAGATATTGCTGGATTGG
oLC8800	Ca*VPS4*+1518-R	CCAATGAGAATTGATAGCGAAGACG
oLC8801	Ca*VPS4*-168-F	TGCGATTTTCAAAGATGTCGG
oLC8802	Ca*VPS4*+1775-R	TCATGGAGAACCAGAAAGACG
oLC8803	Ca*VPS20*-64_KpnI-F	TTGCGGTACCCGAGTTATCAGGAACTGTAATTTGC
oLC8804	Ca*VPS20*+651_ApaI-R	TTGCGGGCCCTTATATGGCAATCGGTTCATG
oLC8805	Ca*VPS20*+651_SacII-F	TTGCCCGCGGCCAACAACATAATTCTATGTGAGTC
oLC8806	Ca*VPS20*+1271_SacI-R	TTGCGAGCTCTATCAAGACCGGGTTAATGAATGC
oLC8807	Ca*VPS20*+203-F	AATCGGTAATCAGTACCACCTTCG
oLC8808	Ca*VPS20*+895-R	GCCCATCAAGGATTTTTACGAGC
oLC8809	Ca*VPS20*-245-F	TATGCTATTGTCGTGGTCTTGC
oLC9720	*CaRAS1prom*-up+TAR	GGTTATTGCAGCTACTGTACTGATTTGATTGATTAATTTATTTGGGCTG A ATCTATCATATCCCGCCGGGCCCTTGAGATGGAGCCGTCAAATATCC
oLC9721	*CaRAS1start*+GFP	GGTTAAAGCGGATTTACCAACACCACCACCTCCAACAACAACTAATTT AT ATTCTCTCAACATCCACCACCACCTTTGTACAATTCATCCATACC

oLC9725	GFP-F	GGCTGACAAACAAAAGAATGG
oLC9726	Ca*RAS1*-R	TGTCCAGCAGTATCTAAAACATCC
oLC9762	Ca*VPS4*_sgRNA+280-F	TATTATTATATGGACCACCAGTTTTAGAGCTAGAAATAGCAAGTTAAAA
oLC9763	Ca*VPS4*_sgRNA+280-R	TGGTGGTCCATATAATAATACAAATTAAAAATAGTTTACGCAAGTC
oLC9764	Ca*VPS4*_pLC49-F	CCACCTCCACCACCACCACTATTACAAATCATATAGACTGATTTAAACT TATAAAACAATACACTTAAGCGGAAACAGCTATGACCATG
oLC9765	Ca*VPS4*_pLC49-R	ACGTTGTTTTAAAAGAGACTACTATAGAAATGAATATTTCTTGAATATAT TTTATTCATTGGCCCCTCTTGTAAAACGACGGCCAG
oLC9767	Ca*VPS20*_sgRNA+308-F	GTTGTGTATGGATTACAACAGTTTTAGAGCTAGAAATAGCAAGTTAAAA
oLC9768	Ca*VPS20*_sgRNA+308-R	TGTTGTAATCCATACACAACCAAATTAAAAATAGTTTACGCAAGTC
oLC9769	Ca*VPS20*_pLC49-F	AACCTACGAGTTATCAGGAACTGTAATTTGCTATCATAATCAATATAGA TCTAATACATTCAAATTGACAGGAAACAGCTATGACCATG
oLC9770	Ca*VPS20*_pLC49-R	TTTTATGTGTATGTAAATGCAAAAATTTATTATTAAGTGTATTATGACT CACATAGAATTATGTTGTTGGGTAAAACGACGGCCAG

Supplemental References:

1. White, T. C. Increased mRNA levels of ERG16, CDR, and MDR1 correlate, with increases in azole resistance in Candida albicans isolates from a patient infected with human immunodeficiency virus. *Antimicrobial Agents and Chemotherapy* **41**, 1482–1487 (1997).
2. Jones, T. *et al.* The diploid genome sequence of Candida albicans. *Proc Natl Acad Sci U S A* **101**, 7329–7334 (2004).
3. Bruno, V. M. & Mitchell, A. P. Regulation of azole drug susceptibility by Candida albicans protein kinase CK2. *Molecular Microbiology* **56**, 559–573 (2005).
4. Perea, S. *et al.* Prevalence of Molecular Mechanisms of Resistance to Azole Antifungal Agents in Candida albicans Strains Displaying High-Level Fluconazole Resistance Isolated from Human Immunodeficiency Virus-Infected Patients. *Antimicrobial Agents and Chemotherapy* **45**, 2676 (2001).
5. Blankenship, J. R. & Heitman, J. Calcineurin is required for Candida albicans to survive calcium stress in serum. *Infection and Immunity* **73**, 5767–5774 (2005).

6. Noble, S. M. & Johnson, A. D. Strains and strategies for large-scale gene deletion studies of the diploid human fungal pathogen Candida albicans. *Eukaryotic Cell* **4**, 298–309 (2005).

7. Braun, B. R. & Johnson, A. D. TUP1, CPH1 and EFG1 make independent contributions to filamentation in Candida albicans. *Genetics* **155**, 57–67 (2000).

8. Whitesell, L. *et al.* Structural basis for species-selective targeting of Hsp90 in a pathogenic fungus. *Nature Communications* **10**, 1–17 (2019).

9. Cowen, L. E. *et al.* Population genomics of drug resistance in Candida albicans. *Proc Natl Acad Sci U S A* **99**, 9284–9289 (2002).

10. Singh, S. D. *et al.* Hsp90 governs echinocandin resistance in the pathogenic yeast Candida albicans via calcineurin. *PLoS Pathogens* **5**, 1000532 (2009).

11. Cormack, B. P. & Falkow, S. Efficient homologous and illegitimate recombination in the opportunistic yeast pathogen Candida glabrata. *Genetics* **151**, 979–987 (1999).

12. Reedy, J. L., Filler, S. G. & Heitman, J. Elucidating the Candida albicans calcineurin signaling cascade controlling stress response and virulence. *Fungal Genetics and Biology* **47**, 107–116 (2010).

13. Heilmann, C. J., Schneider, S., Barker, K. S., Rogers, P. D. & Morschhäuser, J. An A643T Mutation in the Transcription Factor Upc2p Causes Constitutive ERG11 Upregulation and Increased Fluconazole Resistance in Candida albicans. *Antimicrobial Agents and Chemotherapy* **54**, 353 (2010).

14. Sanglard, D., Ischer, F., Marchetti, O., Entenza, J. & Bille, J. Calcineurin A of Candida albicans: Involvement in antifungal tolerance, cell morphogenesis and virulence. *Molecular Microbiology* **48**, 959–976 (2003).

15. Granger, D. L., Perfect, J. R. & Durack, D. T. Virulence of Cryptococcus neoformans: Regulation of capsule synthesis by carbon dioxide. *Journal of Clinical Investigation* **76**, 508–516 (1985).

16. Roemer, T. *et al.* Large-scale essential gene identification in Candida albicans and applications to antifungal drug discovery. *Molecular Microbiology* **50**, 167–181 (2003).

17. Nierman, W. C. *et al.* Genomic sequence of the pathogenic and allergenic filamentous fungus Aspergillus fumigatus. *Nature* **438**, 1151–1156 (2005).

18. Chatterjee, S. *et al.* Draft genome of a commonly misdiagnosed multidrug resistant pathogen Candida auris. *BMC Genomics* **16**, 686 (2015).

19. Shen, J., Guo, W. & Köhler, J. R. CaNAT1, a heterologous dominant selectable marker for transformation of Candida albicans and other pathogenic Candida species. *Infection and Immunity* **73**, 1239–1242 (2005).

20. Veri, A. O. *et al.* Tuning Hsf1 levels drives distinct fungal morphogenetic programs with depletion impairing Hsp90 function and overexpression expanding the target space. *PLoS Genetics* **14**, e1007270 (2018).
