# Supplementary material for: Targeting fungal membrane homeostasis with imidazopyrazoindoles impairs azole resistance and biofilm formation
Source: Nat Commun. 2022 Jun 25;13:3634. doi: 10.1038/s41467-022-31308-1 (PMC9233667; doi:10.1038/s41467-022-31308-1)
Supplement: Supplementary file 2 — Reporting Summary [file 41467_2022_31308_MOESM2_ESM.pdf]

nature portfolio

Corresponding author(s): Leah E. Cowen

Last updated by author(s): Jun 2, 2022

Reporting Summary

Nature Portfolio wishes to improve the reproducibility of the work that we publish. This form provides structure for consistency and transparency in reporting. For further information on Nature Portfolio policies, see our Editorial Policies and the Editorial Policy Checklist.

Statistics

For all statistical analyses, confirm that the following items are present in the figure legend, table legend, main text, or Methods section.

n/a	Confirmed	
☐	☒	The exact sample size (n) for each experimental group/condition, given as a discrete number and unit of measurement
☐	☒	A statement on whether measurements were taken from distinct samples or whether the same sample was measured repeatedly
☐	☒	The statistical test(s) used AND whether they are one- or two-sided<br>*Only common tests should be described solely by name; describe more complex techniques in the Methods section.*
☒	☐	A description of all covariates tested
☐	☒	A description of any assumptions or corrections, such as tests of normality and adjustment for multiple comparisons
☐	☒	A full description of the statistical parameters including central tendency (e.g. means) or other basic estimates (e.g. regression coefficient) AND variation (e.g. standard deviation) or associated estimates of uncertainty (e.g. confidence intervals)
☐	☒	For null hypothesis testing, the test statistic (e.g. F, t, r) with confidence intervals, effect sizes, degrees of freedom and P value noted<br>*Give P values as exact values whenever suitable.*
☒	☐	For Bayesian analysis, information on the choice of priors and Markov chain Monte Carlo settings
☒	☐	For hierarchical and complex designs, identification of the appropriate level for tests and full reporting of outcomes
☒	☐	Estimates of effect sizes (e.g. Cohen's d, Pearson's r), indicating how they were calculated

Our web collection on statistics for biologists contains articles on many of the points above.

Software and code

Policy information about availability of computer code

Data collection	CytExpert was used for flow cytometry data collection. TECAN GENios was used for growth curve assays. Thermo Finnigan TSQ Quantum Ultra Mass Spectrometer was used for sphingolipid quantification. For whole genome sequencing, Bowtie2 (v2.4.0) was used to align sequencing reads with reference genome and the alignment was visualized using Integrative Genomics Viewer. MuTect (v1.1.7) was used to identify unique mutations compared to the parental strain
Data analysis	YMAP (v1.0), GraphPad Prism (v9.3.0), Java TreeView 1.1.3, XFluor4 software for TECAN GENios, TargetLynx Waters v4.1, Volocity, Microsoft Excel (v16.60), NanoAnalyze software (TA instrument)

For manuscripts utilizing custom algorithms or software that are central to the research but not yet described in published literature, software must be made available to editors and reviewers. We strongly encourage code deposition in a community repository (e.g. GitHub). See the Nature Portfolio guidelines for submitting code & software for further information.

Data

Policy information about availability of data

All manuscripts must include a data availability statement. This statement should provide the following information, where applicable:

- Accession codes, unique identifiers, or web links for publicly available datasets
- A description of any restrictions on data availability
- For clinical datasets or third party data, please ensure that the statement adheres to our policy

All data generated or analyzed during this study are included in this published article (and its supplementary information files), or are available from the corresponding author upon reasonable request.

Field-specific reporting

Please select the one below that is the best fit for your research. If you are not sure, read the appropriate sections before making your selection.

☒ Life sciences ☐ Behavioural & social sciences ☐ Ecological, evolutionary & environmental sciences

For a reference copy of the document with all sections, see nature.com/documents/nr-reporting-summary-flat.pdf

Life sciences study design

All studies must disclose on these points even when the disclosure is negative.

Sample size	Sample size was always n=3 or greater when statistical analysis was required in order to adequately assess any variation in the data. All experiments were performed in biological duplicate or greater with little deviation between replicates.
Data exclusions	In flow cytometry experiments, events were excluded from calculation of median fluorescence intensity (MFI) by gating on forward and side scatter parameters to eliminate debris and multi-cell clumps that would skew data. This was based on previous analyses that confirmed the appropriate sizing of yeast cells. Gating removed less than 15% of all acquired events. No other data were excluded from analysis.
Replication	All attempts at replication were successful. Unless othersie notes, all experiments are representative of at least two biological replicates.
Randomization	Randomization was not relevant to the type of experimentation reported. All assays had a quantitative output, rather than qualitative, and therefore, randomization was not required to eliminate user bias.
Blinding	Blinding was not relevant to this study. All assays had quantitative output, rather than qualitative, and therefore, blinding was not required to eliminate user bias.

Reporting for specific materials, systems and methods

We require information from authors about some types of materials, experimental systems and methods used in many studies. Here, indicate whether each material, system or method listed is relevant to your study. If you are not sure if a list item applies to your research, read the appropriate section before selecting a response.

Materials & experimental systems

n/a	Involved in the study	
☒	☐	Antibodies
☐	☒	Eukaryotic cell lines
☒	☐	Palaeontology and archaeology
☐	☒	Animals and other organisms
☒	☐	Human research participants
☒	☐	Clinical data
☒	☐	Dual use research of concern

Methods

n/a	Involved in the study	
☒	☐	ChIP-seq
☐	☒	Flow cytometry
☒	☐	MRI-based neuroimaging

Eukaryotic cell lines

Policy information about cell lines

Cell line source(s)	HepG2 (ATCC HB-8085); Human HEK 293T cells were obtained from ATCC (ATCC Cat # CRL-3216).
Authentication	Cell line was not authenticated as specific tissue of origin was non-critical to validity of results reported.
Mycoplasma contamination	All cell lines tested negative for mycoplasma contamination by PCR-based detection.
Commonly misidentified lines (See ICLAC register)	No commonly misidentified cell lines were used.

Animals and other organisms

Policy information about studies involving animals; ARRIVE guidelines recommended for reporting animal research

Laboratory animals	Spraw Daley (purchased from Envigo), male rats, 10 weeks old for the rat catheter experiments.
Wild animals	No wild animals were used in this study.
Field-collected samples	No field-collected animals were used in this study.

Ethics oversight	Rat catheter biofilm animal procedures were approved by the Institutional Animal Care and Use Committee at the University of Wisconsin-Madison according to the guidelines of the Animal Welfare Act, The Institute of Laboratory Animals Resources Guide for the Care and Use of Laboratory Animals, and Public Health Service Policy. The approved animal protocol number is DA0031.

Note that full information on the approval of the study protocol must also be provided in the manuscript.

Flow Cytometry

Plots

Confirm that:

- [x] The axis labels state the marker and fluorochrome used (e.g. CD4-FITC).
- [x] The axis scales are clearly visible. Include numbers along axes only for bottom left plot of group (a 'group' is an analysis of identical markers).
- [x] All plots are contour plots with outliers or pseudocolor plots.
- [x] A numerical value for number of cells or percentage (with statistics) is provided.

Methodology

Sample preparation	For Cdr1-GFP experiments, CaLC7116 was subcultured in YPD medium in 3 mL at an OD600 0.2 for 3 hours with agitation. Indicated compounds were added and cells were incubated for an additional 30 minutes. Cells were then pelleted by centrifugation at 3,000 xg for 5 minutes and washed 2x in PBS. Cells were added to a flat bottom, transparent, 96-well plate (Beckman Coulter). Each sample was run using the CytExpert Software (version 2.4) until ~20,000 events had been recorded. Populations were gated to exclude debris and doublets, and the median value was taken for each sample.
Instrument	CytoFLEX S (Beckman Coulter)
Software	CytExpert Software (v2.4)
Cell population abundance	The cell population post-sort was approximately 80% of the population.
Gating strategy	Populations were gated to exclude debris and doublets, and the median value was taken for each sample. (See Supplementary Fig. 2a).

- [x] Tick this box to confirm that a figure exemplifying the gating strategy is provided in the Supplementary Information.
